# Identifying fundamental goals of childbirth care for women with higher body weight in Swiss maternity care: an embedded mixed methods multi-stakeholder study

**DOI:** 10.1136/bmjopen-2024-086409

**Published:** 2025-07-22

**Authors:** Carmen Wyss, Jennifer Inauen, Judit Lienert, Evelyne M Aubry

**Affiliations:** 1 Applied Research and Development, Division of Midwifery, Department of Health Professions, Bern University of Applied Sciences, Bern, Switzerland; 2 Graduate School for Health Sciences, University of Bern, Bern, Switzerland; 3 Department of Health Psychology and Behavioral Medicine, Institute of Psychology, University of Bern, Bern, Switzerland; 4 Decision Analysis Group, Department of Environmental Social Sciences, Swiss Federal Institute of Aquatic Science and Technology (Eawag), Duebendorf, Switzerland

**Keywords:** Body Mass Index, Decision Making, Methods, Obesity, OBSTETRICS, Quality in health care

## Abstract

**Abstract:**

**Objectives:**

The first objective of the study was to identify fundamental goals of childbirth care for women with higher body weight to inform decision-making processes by drawing on multi-stakeholder insights. The second objective included evaluating the method used to support these stakeholders in generating a more diverse set of fundamental goals.

**Design:**

Using an embedded mixed methods design, we engaged key stakeholders in two group workshops and 11 individual interviews to identify goals considered fundamentally important for achieving the best possible childbirth care. Participants individually brainstormed childbirth care goals, selected additional goals from a pre-established masterlist and discussed them. Thereafter, participants rated the goals’ perceived importance for decision-making on a Likert scale. We thematically analyzed the goals and mapped them onto a means-ends network to identify and refine a set of fundamental goals. Methodological evaluations involved descriptive statistics and non-parametric testing.

**Setting:**

Swiss maternity care.

**Participants:**

21 stakeholders, including seven women with a preconceptional body mass index ≥ 30 kg/m^2^, nine midwives, and five obstetricians experienced in maternity care for women with higher body weight.

**Results:**

We identified eight fundamental goals of childbirth care: low maternal and neonatal complication rates, physiological childbirth processes, positive initiation of bonding and breastfeeding, positive psychosocial care experience, low physical strain for care providers, low resource use in care setting, and low direct costs of childbirth care. Individual participants generated more diverse fundamental goals on average by combining individual brainstorming with goal selection from a masterlist than by brainstorming alone. A theoretical maximum of six participants captured all eight fundamental goals.

**Conclusions:**

The fundamental goals provide a framework for benchmarking decisions to improve childbirth care for women with higher body weight. Additionally, individual brainstorming combined with goal selection from a masterlist appears to be a useful method for generating a diverse set of fundamental goals in a healthcare context, even with relatively few participants.

**STRENGTHS AND LIMITATIONS OF THIS STUDY**

- The study employed an exploratory approach using an embedded mixed methods design to identify fundamental goals of childbirth care for women with higher body weight, adapting an established goal-elicitation method from decision analysis to the healthcare context.
- The involvement of multiple key stakeholders in the goal-elicitation process ensured diverse perspectives from both women with higher body weight and their maternity care providers.
- Methodological evaluations were conducted to assess the applicability and robustness of the goal-elicitation method within healthcare research.
- The fundamental goals of childbirth care will need to be validated and potentially expanded to include diverse socioeconomic and cultural contexts, different healthcare systems, and the general birthing population to assess the transferability of the findings beyond the specific setting of this study.

## Introduction

 Childbirth care decisions for women with higher body weight can be complex due to unique challenges they may face during childbirth. Literature has highlighted the increased likelihoods of obstetric complications, medical interventions, and adverse events for both mothers and their children associated with higher maternal body weight (preconceptional body mass index (BMI) ≥ 30 kg/m^2^).^1^,^4^

Existing guidelines on maternity care for women with higher body weight^5^,^7^ have predominantly focused on biomedical aspects to improve outcomes. Concurrently, the importance of recognizing the psychosocial dimension of care^8 9^ and the utilization of resources^8^ has also been emphasized. For instance, weight stigma, a prevalent issue in the care of women with higher body weight, may affect their psychosocial experience,^10 11^ impact the quality of care,^12^ and lead to poorer biopsychosocial health outcomes.^13^,^15^ Additionally, addressing resource utilization is likely relevant as different healthcare settings can vary in their capacities to handle increased resource demands linked to higher body weight.^8^ Moreover, financial burdens experienced by women and their families^8^ might shape care decisions. Multidimensional values and priorities that may influence childbirth care decisions should therefore be explored in collaborative discussions between women and their maternity care providers to promote tailored, high-quality care.

In order to provide high-quality childbirth care to individuals and populations, we should strive for achieving the goals that align with the women’s needs and preferences and the best available scientific evidence.^16^ This approach emphasizes a person-centered, evidence-based perspective and seeks to improve perinatal outcomes within the broader context of care. Recognizing multiple, potentially conflicting, fundamental goals is thereby essential to guiding decisions towards tailored childbirth care. These overarching goals are informed by the underlying values and priorities^17 18^ of women and their maternity care providers, reflecting what is pivotal to achieve during a care episode.^19^ Identifying and understanding such fundamental goals provides a comprehensive framework for selecting the best possible childbirth care option in a given decision situation. In decision analysis, these goals are often referred to as decision objectives^20^ or decision criteria,^21^ although the terminology may vary.

The significance of identifying care goals in healthcare decision-making is well acknowledged^18 19^ as incomplete representations of values and priorities can profoundly affect care decisions and outcomes. Involving key stakeholders is thereby consistent with contemporary ethical and moral standards in healthcare^22 23^ and enhances the legitimacy and acceptability of decisions.^24^ Ongoing multi-stakeholder research aims to establish a core outcome set for trials evaluating interventions in pregnant women with higher body weight.^25^ However, we found no studies that explicitly examined fundamental childbirth care goals to inform decision-making specifically for women with higher body weight nor for the general birthing population. Explicit knowledge of such goals could structure the biopsychosocial complexities of navigating childbirth care for women with higher body weight and facilitate goal-oriented deliberation throughout decision-making processes.^24^

For complex decisions, stakeholders may struggle to articulate their goals without support, making it critical to help them identify what is fundamentally important to achieve.^26^,^28^ Research by Bond *et al*^26 27^ and Haag *et al*^28^ has demonstrated that combining brainstorming with selecting relevant goals from a pre-established masterlist can generate more comprehensive sets of goals in general decision analysis studies and public environmental planning. Notably, even limited numbers of participants have thereby been shown to capture the complete set of goals.^28^ We hypothesized that these findings may generalize to the healthcare context. While this has not yet been empirically validated, it could inform future efforts in identifying healthcare goals.

To address these knowledge gaps, the study focused on the following three research questions: (1) What are the fundamental goals of childbirth care for women with higher body weight? (2a) Can a combination of brainstorming and goal selection from a masterlist help stakeholders generate a more diverse set of fundamental goals in a healthcare context? (2b) How many participants are required to fully capture the fundamental goals using this method? Therewith, the first objective of the study was to identify a diverse set of fundamental goals of childbirth care for women with higher body weight to inform decision-making processes by drawing on multi-stakeholder insights. The second objective included evaluating the masterlist method to support these healthcare stakeholders in generating a more diverse set of fundamental goals.

## Methods

### Research approach and study design

Given the limited prior research on our study topic, we employed an exploratory approach using an embedded mixed methods design. The exploratory approach allowed to systematically integrate qualitative and quantitative methods^29^ to gain new insights into fundamental goals of childbirth care for women with higher body weight and to evaluate the masterlist method for supporting goal generation in a healthcare context. The embedded mixed methods design well-suited our study as the interrelated research questions required the combination of different but complementary types of data.^30^ To identify goals considered fundamentally important for achieving the best possible childbirth care, we conducted group workshops and individual interviews with key stakeholders. This qualitative process was guided by value-focused thinking according to Keeney.^31^ We aimed to uncover the stakeholders’ underlying values and priorities, rather than centering decision-making on selecting among predefined options, as in conventional ‘alternative-focused thinking’.^31^ The quantitative component, which assessed the masterlist method, was embedded within the qualitative process.^30^ While each component addressed separate research questions, the quantitative evaluation was inherently dependent on the qualitative identification of the fundamental care goals.^30^ The embedded mixed methods design therefore facilitated a better understanding of both the fundamental goals of childbirth care for women with higher body weight and the usefulness of the masterlist method for generating diverse healthcare goals.

### Patient and public involvement

Women with higher body weight and their maternity care providers were actively engaged as key stakeholders to identify goals of childbirth care. However, the study did not involve patients or members of the public in its design, conduct, reporting, or dissemination plans.

### Stakeholder eligibility for participation and recruitment

Eligible participants included women who were pregnant or had given birth in the past 10 years with a preconceptional BMI ≥ 30 kg/m^2^. Midwives and obstetricians were eligible if they had at least two years of self-reported experience in childbirth care for women with higher body weight. Additionally, participants needed a good command of (Swiss) German and access to an internet-connected device, as in-person data collection was primarily conducted online due to the SARS-CoV-2 pandemic.

To recruit participants, we invited five public and private obstetric clinics, three birth centers and eight independent private (group) practices in the German-speaking regions of Switzerland to share our participation call with eligible patients and care providers. The call was also disseminated through online Swiss obesity and body respect platforms, social media, and the researchers’ professional networks.

Interested stakeholders could contact us via email or telephone, or directly register online for a group workshop or an individual interview. We provided detailed study information, asking participants to review it carefully before signing and returning the consent form. They could seek clarification via email, telephone, or in-person before data collection. All participants provided written informed consent.

### Development of the masterlist

We adapted the masterlist method from decision analysis^26^,^28^ as previous research has shown that stakeholders may overlook important goals for decision-making when simply being asked.^26^,^28^ This method was applied in group workshops and individual interviews to systematically identify diverse goals of childbirth care for women with higher body weight. Before in-person data collection, we therefore developed a masterlist of potential goals of childbirth care, following the methodology of Haag *et al.*^28^ This masterlist was informed by literature addressing quality standards, quality indicators, core outcome sets, standardized outcomes, patient-reported outcomes, and other reported outcome measures for pregnancy and childbirth.^89 16 32^,^39^ While not limited to a specific population, the literature included considerations for childbirth care for individuals with higher body weight. The resulting masterlist consisted of 18 preliminary goals potentially relevant to the decision context (online supplemental material S1).

### Data collection from participants

Between September 2021 and February 2022, we conducted two group workshops and 11 individual interviews with a total of 21 stakeholders. The two workshops with five participants each (three midwives and two obstetricians; five midwives) and seven interviews (five women with higher body weight, two obstetricians) were held online. We conducted the remaining four interviews face-to-face (two women with higher body weight, one midwife, one obstetrician). The workshop and interview guide was adapted from Beutler *et al*^40^ and is available in the online supplemental material S2. Two researchers facilitated the workshops, with CW guiding the data collection steps and moderating discussions, while EA took comprehensive notes. CW conducted the interviews. The data collection process involved five steps, as detailed in table 1:

After an introduction (step 1), participants individually brainstormed goals of childbirth care for women with higher body weight (step 2). They then compared their goals to the masterlist of 18 potential care goals that we had previously developed (online supplemental material S1), and identified important new goals from the masterlist (step 3). Thereafter, participants discussed collectively or elaborated individually on the goals, delving into underlying values, modifying and categorizing goals (step 4). Within one to three days of the workshops and interviews, we sent participants an online questionnaire to quantitatively rate the generated goals’ perceived importance for decision-making on a Likert scale from 0 (not important) to 4 (essential)^28^ (step 5). The workshops lasted 120 min, the interviews 45 to 90 min. The time to complete the questionnaire ranged from 5 to 20 min.

**Table 1:** Data collection from participants and data analysis for identifying fundamental goals

	Step	What?	Why?	How?
Data collection	1	Introduction	Familiarize participants with the topic, the importance of their contribution, what to expect, and ensure informed consent	Participants were informed about the study’s topic, rationale, purpose, and data collection process, and had the opportunity to ask questions. They provided informed consent to participate and to be video/audio recorded.
	2	Initial generation of goals	Allow participants to generate goals without influence by other participants or facilitators	Individual brainstorming session was held around the question ‘What goals should be achieved through childbirth care for women with higher body weight?’ Participants silently wrote down goals they considered important for themselves or other stakeholders.
	3	Completion of goals using the masterlist	Support participants in articulating what is important to them^26^,^28^	Participants compared their *self-generated* goals from the brainstorming with the goals on the masterlist (online supplemental material S1). They selected (ie, *recognized*) additional new goals from the masterlist they considered important. All goals were recorded and displayed virtually or on paper for participants to confirm or revise as needed.
	4	Clarification of goals	Ensure clarity of the goals and understand why the goals are important to participants	Participants discussed (in workshops) or individually elaborated (in interviews) their understanding of the goals and explored their underlying values with the opportunity to modify and categorize goals as needed.^79^ New goals that emerged during group discussions in workshops were recorded for thematic analysis. Finally, all goals were summarized, and participants were asked to approve their content and completeness.
	5	Rating of goals	Individually prioritize the goals based on what participants perceive to be most important to inform decision-making	Participants rated the goals’ perceived importance for decision-making on a Likert scale (0=not important; 1=rather not important; 2=rather important; 3=important; 4=essential; with the option ‘don’t know‘)^28^ and provided basic sociodemographic characteristics via an online questionnaire.
Data analysis	6	Extraction of unique goals	Prepare non-redundant and specific data for analysis	Goals from all workshops and interviews with the same underlying meaning were merged to avoid redundancy.^80^ Items that were no goals^44^ and goals out of context (eg, care options, family policy goals) were excluded.
	7	Thematic analysis of goals	Develop a framework of childbirth care goals	CW and EA independently analyzed the goals thematically,^43^ developing a goal framework based on insights from patterns in the collected data and participants’ categorization (online supplemental material S3). Results were compared and discussed at a two-day meeting, and disagreements resolved as necessary.
	8	Identification of fundamental goals	Make the underlying values explicit for decision-making^31^	Goals were mapped onto a means-ends network to qualitatively explore their interrelationships and to distinguish between *means goals* (ie, intermediate states) and the underlying *fundamental goals* at the core of the decision context (ie, the important *ends goals*)^42 44 45^ (online supplemental material S4). Identification and refinement of the final set of fundamental goals was derived from the goal framework (step 7) and the means-ends network through an iterative process within the research team.
	9	Evaluation of the perceived importance	Explore potential goal prioritization among participants	Perceived importance was assessed by average ratings for the fundamental goals. Ordinal rating data were treated as cardinal to construct importance indices, despite the risk of range insensitivity bias in this simple form of relevance assessment.^72^

Data collection steps were adapted from Bond *et al*^26 27^ and Haag *et al.*^28^

### Data analysis for identifying fundamental goals

We analyzed the data to distill a set of distinct fundamental care goals that meet the requirements of a good set of goals for decision analysis^41^: *Completeness* (encompassing all values fundamentally important to stakeholders), *conciseness* (capturing all relevant aspects in as few goals as possible), *understandability* (ensuring clarity for stakeholders), *non-redundancy* (preventing overlaps of goals and/or double-counting), *measurability* (achieving the goals can be measured by indicators), and *preferential independence* (ensuring that the preference for one goal does not depend on another, if possible).^20 42^ This analytical process has been described as ‘perhaps the most challenging and time-consuming phase’ in decision analysis.^42^ It required several steps to derive a set of fundamental childbirth care goals (second part of table 1):

First, we created a list of unique goals from all collected data (step 6) and analyzed the goals using a thematic approach for creating a goal framework^43^ (online supplemental material S3) (step 7). The goals were graphically mapped onto a network of *means goals* (ie, intermediate states in achieving *ends goals*) and underlying *fundamental goals* (ie, important *ends goals* in the specific decision context). To explore interrelationships between the goals and to make underlying values explicit, we asked: ‘Why is that goal important in the decision context?’ (online supplemental material S4).^42 44 45^ A final set of fundamental goals was then refined from the goal framework and the means-ends network (step 8). Finally, we analyzed the perceived importance data by calculating average ratings for the fundamental goals (step 9).

### Data analysis for evaluating the masterlist method

To evaluate whether combining brainstorming with goal selection from a masterlist can help stakeholders in a healthcare context to generate more diverse fundamental goals,^26^,^28^ the fundamental goals in this study were categorized into either *self-generated* or *recognized* by individual participants.^26 28^ We defined a fundamental goal as *self-generated* if participants identified at least one goal assigned to this fundamental goal during individual brainstorming without support of the masterlist. Participants were considered to have *recognized* a fundamental goal if they selected it from the masterlist without self-generating any other goals assigned to the same fundamental goal. Using these definitions, we evaluated the number of distinct fundamental goals identified by each participant, distinguishing between self-generated fundamental goals during brainstorming and those selected (ie, recognized) from the masterlist. The proportion of fundamental goals identified by each participant and the average perceived importance of these fundamental goals were calculated based on whether they were self-generated or recognized. To assess whether the masterlist supported the generation of more distinct fundamental goals, we used the Wilcoxon signed-rank test to compare the total number of fundamental goals identified with the number of self-generated fundamental goals. We examined differences in the average perceived importance of self-generated versus recognized fundamental goals using the same statistical test. Since only a subset of participants (n=10, 48%) attended workshops and had the opportunity to develop new goals during group discussions (table 1, step 4), we did not analyze the goals generated during this phase separately.

A saturation analysis inspired by Haag *et al*^28^ was conducted to determine the number of participants required to capture all fundamental goals identified in this study. For this, we assessed how many new fundamental goals each additional participant theoretically contributed. To estimate the minimum number of participants, we started with those who generated the most distinct fundamental goals, ordering them by their maximum number of additional fundamental goals they contributed. We then determined how many participants were required to capture all fundamental goals. Conversely, to estimate the maximum number of participants, we followed the opposite approach — starting with those who generated the fewest distinct fundamental goals and counting the number of participants required to capture all fundamental goals when ordered by their minimum contribution. To cross-check our estimates, we adopted a mathematical formula^46^ for goal detection as a function of the participant count: $identified\left(i\right)=N\left(1-{\left(1-\lambda \right)}^{i}\right)$, where *identified(i*) represents the number of fundamental goals identified by *i* participants, *N* is the total number of fundamental goals identified in the study, and λ is the average proportion of all fundamental goals identified by an individual participant. We used Stata/MP 15.1^47^ for statistical analysis and graphing.

## Results

### Study participants

Seven women with higher body weight, nine midwives, and five obstetricians participated in the study (table 2). All reported being female and aged between 30 and 64 years (mean=40.0 years). The women with higher body weight had given birth to at least one child, and three were pregnant at the time of the interview. Their preconceptional BMI ranged between 30.5 and 44.1 kg/m^2^ (mean=36.0 kg/m^2^). The professional experience of the maternity care providers ranged from three to 35 years (mean=15.3 years).

**Table 2:** Participants’ characteristics

Characteristic (m)	Statistic	All participants	Women with higher body weight	Midwives	Obstetricians
		(N=21, 100%)	(n=7, 33.3%)	(n=9, 42.9%)	(n=5, 23.8%)
Age (0)	Range (mean; median)	30.0–64.0 (40.0; 37.0)	32.0–40.0 (36.4; 37.0)	31.0–64.0 (41.9; 36.0)	30.0–59.0 (41.4; 40.0)
Self-reported gender (0)					
Female	n (%)	21 (100.0)	7 (100.0)	9 (100.0)	5 (100.0)
Self-reported ethnic group (0)					
White	n (%)	21 (100.0)	7 (100.0)	9 (100.0)	5 (100.0)
BIPoC	n (%)	0 (0.0)	0 (0.0)	0 (0.0)	0 (0.0)
Civil status (0)					
Unmarried	n (%)	8 (38.1)	2 (28.6)	2 (22.2)	4 (80.0)
Married/registered partnership	n (%)	13 (61.9)	5 (71.4)	7 (77.8)	1 (20.0)
Number of children (0)					
No children	n (%)	5 (23.8)	0 (0.0)	3 (33.3)	2 (40.0)
One child	n (%)	7 (33.3)	5 (71.4)	1 (11.1)	1 (20.0)
Two or more children	n (%)	9 (42.9)	2 (28.6)	5 (55.6)	2 (40.0)
BMI (in kg/m^2^) (2)	Range (mean; median)	20.9–44.1 (28.7; 25.4)	30.5–44.1 (36.0; 33.1)	21.1–35.2 (25.3; 24.1)	20.9–24.3 (22.1; 21.0)
Highest educational attainment (0)					
Compulsory education	n (%)		0 (0.0)		
Upper secondary education	n (%)		0 (0.0)		
Tertiary education	n (%)		7 (100.0)		
Net-equivalent monthly household income (in CHF) (0)	Range (mean; median)		2222–7777 (5170; 5555)		
Workplace* (0)					
Hospital	n (%)			6 (66.7)	4 (80.0)
Birth center	n (%)			1 (11.1)	0 (0.0)
Self-employed	n (%)			3 (33.3)	1 (20.0)
Professional experience in years (0)	Range (mean; median)			5.5–35.0 (16.9; 11.0)	3.0–25.0 (12.4; 10.0)

* Multiple answers possible.

BIPoC, Black, Indigenous, People of Colour; BMI, body mass index; (m), number of missing values.

### Fundamental goals of childbirth care

**Figure 1:**
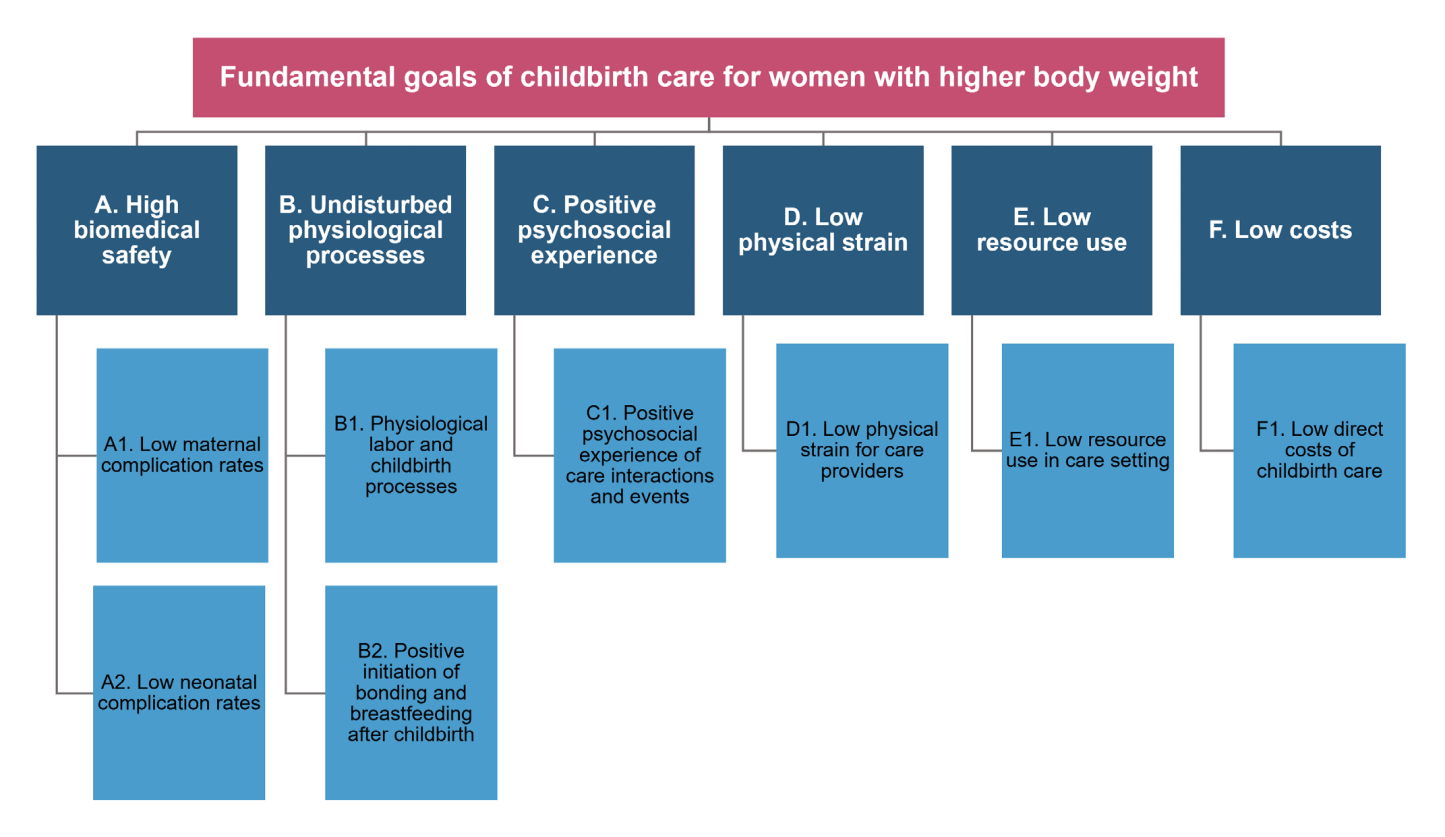
Final set of fundamental goals of childbirth care for women with higher body weight.

**Figure 2:**
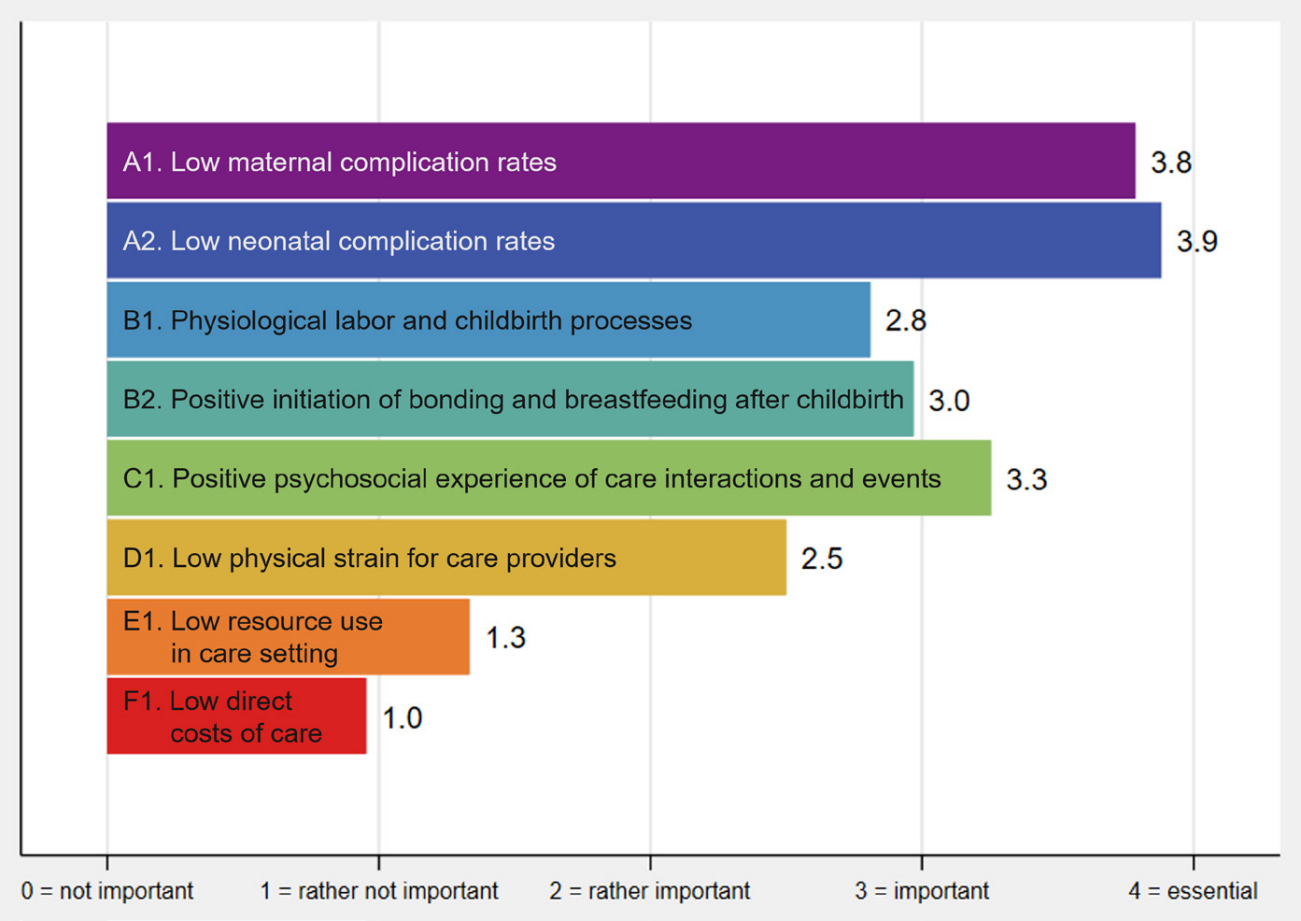
Average perceived importance of fundamental goals for decision-making. Perceived importance for decision-making rated on an ordinal Likert scale^28^ and averaged across participants: 0=not important, 1=rather not important, 2=rather important, 3=important, 4=essential (cf. table 1, step 5). Ordinal rating data were treated as cardinal to construct importance indices for an initial exploration of potential goal prioritization among participants.

We identified a final set of eight fundamental goals of childbirth care for women with higher body weight (figure 1). Details on these fundamental goals are given in table 3. Participants perceived *low maternal and neonatal complication rates* (A1 and A2) to be most important for decision-making on average, followed by *positive psychosocial experience of care interactions and events* (C1) (figure 2).

**Table 3:** Descriptions of fundamental goals and generation by individual participants

Fundamental goals	Descriptions of fundamental goals informed by discussions*	Masterlist goals	Additional goals generated in workshops and interviews	Generated by individual participants (N=21)
A1. Low maternal complication rates	Ensuring the safety of mothers with a focus on minimizing complications of labor and childbirth (eg, postpartum hemorrhage, higher grade birth injury, postpartum infection, obstetric wound complication, thromboembolic event, maternal death).	Low maternal morbidity; low maternal mortality	Vital maternal state; prevention of maternal complications and accidents	20 (95%) Self-generated: 17 Recognized: 3
A2. Low neonatal complication rates	Ensuring the safety of newborns with a focus on minimizing complications of labor and birth (eg, neonatal adaptation problems, acidosis, birth trauma, postnatal infection, neonatal death).	Low neonatal morbidity; low neonatal mortality	Vital neonatal state	19 (90%) Self-generated: 16 Recognized: 3
B1. Physiological labor and childbirth processes	Valuing women’s natural ability to give birth and promoting physiological labor and childbirth processes (eg, spontaneous onset and progression of labor, spontaneous vaginal birth). Interventions that interfere with physiological processes should be avoided (eg, induction of labor, augmentation of labor, anesthesia, episiotomy, instrumental vaginal birth, cesarean birth).	Undisturbed physiological labor processes	Physiological childbirth process; physiological vaginal birth; focus on physiology and on ‘what is going well’; avoidance of cesarean birth; no unnecessary interventions on the woman	16 (76%) Self-generated: 12 Recognized: 4
B2. Positive initiation of bonding and breastfeeding after childbirth	Promoting the physiological initiation of bonding and breastfeeding (if possible and desired) immediately after childbirth (eg, by newborn/parent skin-to-skin contact, early initiation of breastfeeding). Interventions that interfere with early bonding and breastfeeding processes should be avoided (eg, separation of newborn and mother/parent, early formula feeding).	Good mother-child-attachment	Immediate bonding; successful/positive breastfeeding initiation; no unnecessary interventions on the newborn; father/partner-child-attachment	18 (86%) Self-generated: 10 Recognized: 8
C1. Positive psychosocial experience of care interactions and events	Enabling a psychosocial experience of care interactions and events during labor and childbirth that makes the women feel safe, supported, in control, and respected at all times,^59^ by creating a safe and supportive setting, making care person-centered, and treating each woman with respect.	High satisfaction with care; positive childbirth experience; safe care environment; well-informed woman; involvement of woman in decisions; dignity; confidentiality; privacy; no stigmatization and discrimination	Good rapport and level of trust; physical and emotional support; appropriate involvement of childbirth companion; focus on the woman’s individuality; taking the woman seriously; responsiveness to needs; shared decision-making; sense of self-responsibility; self-determined childbirth; power of decisions with parents; realistic and honest information; sensitive and balanced communication; positive anticipation of childbirth; no sole focus on body weight; unprejudiced care; undogmatic care; discrimination-sensitivity; unbiased and respectful language	21 (100%) Self-generated: 21 Recognized: 0
D1. Low physical strain for care providers	Minimizing physical strain and risk of musculoskeletal symptoms for maternity care providers (eg, due to heavy lifting, strenuous postures).	–	Low physical strain for care providers	1 (5%) Self-generated: 1 Recognized: -
E1. Low resource use in care setting	Using resources of the care setting consciously by preventing situations that require intensified monitoring and interventions, and thus increased levels of staffing, equipment, and infrastructure (eg, due to complications, anesthesia, operating room time, maternal/neonatal intensive care unit admission).	Low need of staffing; low need of equipment and infrastructure	–	7 (33%) Self-generated: 4 Recognized: 3
F1. Low direct costs of childbirth care	Managing direct monetary costs of labor and childbirth care efficiently to provide high-quality care without unnecessary financial burden to systems and individuals.	Low costs	–	11 (52%) Self-generated: 10 Recognized: 1

* cf. table 1, steps 4 and 8. Self-generated: Participants self-generated at least one goal during the individual brainstorming that was assigned to this fundamental goal. Recognized: Participants selected at least one corresponding goal from the masterlist and did not self-generate any other goals that were assigned to this fundamental goal (cf. table 1, step 3).

### Generation of fundamental goals

Individual participants generated an average of 5.38 (range: 2 to 7) distinct fundamental goals through individual brainstorming and with masterlist support, representing 67.26% (range: 25.00% to 87.50%) of the total eight fundamental goals in our study (table 4). Thereof, they self-generated 4.33 (range: 2 to 7) distinct fundamental goals in individual brainstorming, and they selected (ie, recognized) 1.05 (range: 0 to 5) fundamental goals from the masterlist on average. Participants thus self-generated 81.88% (range: 28.57% to 100%) of their fundamental goals in individual brainstorming, while they recognized 18.12% (range: 0% and 71.43%) of their fundamental goals exclusively with masterlist support. The total number of fundamental goals individually generated by participants in brainstorming and with masterlist support was on average significantly higher than the number of self-generated fundamental goals in brainstorming alone (5.38 vs 4.33; p<0.05). The average perceived importance of self-generated fundamental goals and those recognized from the masterlist did not differ significantly (3.17 vs 2.80; p≥0.05).

**Table 4:** Number, proportion, and perceived importance of fundamental goals identified by participants

All participants (N=21)	In total	Self-generated	Recognized
	mean (range)	mean (range)	mean (range)
Number of fundamental goals each participant identified (n, max.=8)	5.38 (2–7)	4.33 (2–7)	1.05 (0–5)
Proportion of their fundamental goals participants individually self-generated or recognized (%)	–	81.88 (28.57–100.00)	18.12 (0.00–71.43)
Proportion of the complete set of fundamental goals participants individually self-generated or recognized (%)	67.26 (25.00–87.50)	54.17 (25.00–87.50)	13.10 (0.00–62.50)
Average perceived importance of fundamental goals (0-4)*	–	3.17 (1.85–3.97)	2.80 (1.50–4.00)

* Perceived importance for decision-making rated on an ordinal Likert scale^28^ and averaged for fundamental goals that were self-generated or recognized by participants: 0=not important, 1=rather not important, 2=rather important, 3=important, 4=essential (cf. table 1, step 5). Self-generated: Participants self-generated at least one goal during the individual brainstorming that was assigned to this fundamental goal. Recognized: Participants selected at least one corresponding goal from the masterlist and did not self-generate any other goals that were assigned to this fundamental goal (cf. table 1, step 3).

### Number of participants required to capture the fundamental goals

**Figure 3:**
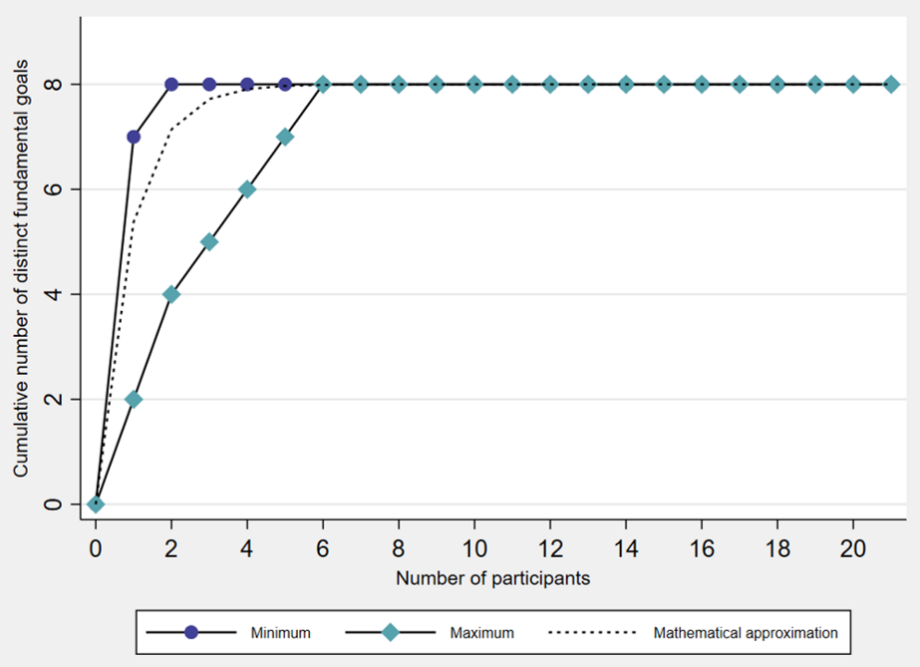
Number of participants required to capture the complete set of fundamental goals. Saturation analysis inspired by Haag *et al.*^28^ Minimum: Cumulative number of eight distinct fundamental goals was reached after two participants. Maximum: Cumulative number of eight distinct fundamental goals was reached after six participants. Mathematical approximation^46^: One participant would theoretically have identified 67.3% of the eight distinct fundamental goals, two participants 89.3%, three participants 96.5%, four participants 98.9%, five participants 99.6%, and six participants 99.9% of the eight distinct fundamental goals in our study.

The saturation analysis indicated that a minimum of two participants and a maximum of six participants would have been sufficient to capture the complete set of eight fundamental goals of childbirth care for women with higher body weight in our study (figure 3). The mathematical approximation showed that one participant would theoretically have identified 67.3% of the eight distinct fundamental goals, two participants 89.3%, three participants 96.5%, four participants 98.9%, five participants 99.6%, and six participants 99.9% of the eight distinct fundamental goals.

## Discussion

Our study provides empirical evidence on multidimensional fundamental goals of childbirth care for women with higher body weight. These goals encompass low maternal and neonatal complication rates, physiological childbirth processes, positive initiation of bonding and breastfeeding, positive psychosocial care experience, low physical strain for care providers, low resource use in care setting, and low direct costs of childbirth care (figure 1; table 3). This fundamental goal set may encourage value-focused deliberation throughout decision-making processes and serve as a framework for finding the most appropriate option to ensure tailored, high-quality childbirth care. In addition, individual brainstorming combined with goal selection from a pre-established masterlist appears to be a more useful method for generating a diverse set of fundamental goals in a healthcare context than brainstorming alone (table 4). This was even the case with relatively few participants in our study (figure 3). These findings reinforce the suitability of the masterlist method (table 1) to guide future efforts in identifying healthcare goals. In the following, we discuss the study’s results in more detail, along with their relevance to clinical practice and research.

### Fundamental goals of childbirth care for women with higher body weight

In this study, we identified a diverse set of fundamental goals of childbirth care for women with higher body weight to inform decision-making processes by drawing on multi-stakeholder insights. Our findings largely align with the well-established notion and the high consensus about the pillars of high-quality childbirth care at a general population level.^16^ However, our study emphasizes the critical importance of these goals in the context of childbirth care for women with higher body weight, given their increased likelihoods of obstetric complications, medical interventions, adverse maternal and neonatal outcomes, and higher resource utilization.^1^,^410 48^ Explicitly addressing multidimensional goals in decision-making may therefore be particularly essential for improving childbirth care and outcomes for women with higher body weight from a biopsychosocial health and resource sustainability perspective.

Biomedical safety has long been an undebated priority in childbirth care, with substantial efforts directed towards reducing morbidity and mortality among mothers and their newborns.^16^ Our findings further underscore the significance of achieving *low maternal and neonatal complication rates* in women with higher body weight and their children, as they may face higher risks of adverse biomedical outcomes compared with women with a BMI < 30 kg/m^2^.^1^,^4^ Concurrently, we found that participants also highly valued *undisturbed physiological processes*. This could be related to the tendency in obstetrics to label women with higher body weight as generally ‘high-risk’,^52^,^55^ which can significantly impact intrapartum care management.^52^ Certain situations may warrant increased medical attention and require interventions to ensure the safety of mother and child. However, detailed surveillance aimed at detecting ‘abnormalities’^56^ and actively managing labor in all women can lead to overmedicalization, result in unnecessary interventions,^57^ and interfere with labor physiology.^58^ Acknowledging potential risks associated with higher maternal body weight while focusing care on what is explicitly going well may emphasize physiological processes and increase the woman’s confidence in her ability to give birth.^58^ Correspondingly, all participants in our study identified *positive psychosocial experience of care interactions and events* as an important fundamental goal of childbirth care for women with higher body weight. This finding is consistent with the concept of experience of care, which has been increasingly recognized in recent years as a critical element of high-quality childbirth care for all birthing individuals.^39^ Our findings thereby reinforce Leinweber *et al*’s definition of a positive childbirth experience as ‘a woman’s experience of interactions and events directly related to childbirth that made her feel supported, in control, safe, and respected’.^59^ Particularly, *feeling respected* appeared to be a pivotal aspect of the experience for women with higher body weight in our study. This corresponds to previous research showing that disrespectful care related to body composition is unfortunately common for women in maternity services.^10 50 55 60^ Maternity care providers may have less positive attitudes towards caring for women with higher body weight,^11^ which may manifest in stigmatizing behaviors.^1355 61^,^64^ Weight stigma can also undermine a woman’s sense of support, safety, and control during childbirth. Moreover, stigmatization may have tangible consequences beyond the time of childbirth such as diminished mental health,^13 14^ poorer health behaviors,^13^ and decreased uptake^13^ or even avoidance^15^ of healthcare. In contrast, this study highlights the value of experiencing safe, supportive, person-centered, and respectful interactions to high-quality childbirth care for women with higher body weight.

Our findings also indicate the need to consider goals on resource sustainability for individuals, institutions, and the healthcare system when discussing appropriate childbirth care for women with higher body weight. One of these values that was reflected in our study was *low physical strain for care providers*. Childbirth care provision often involves strenuous activities such as heavy lifting and awkward postures that can lead to musculoskeletal symptoms. These are highly prevalent among midwives, possibly affecting their activities, and leading to absenteeism or even leaving the profession.^65^ Previous studies have similarly reported of occupational health issues in providing maternity care to women with higher body weight, thus acknowledging the need for adequate equipment and service planning.^66 67^ Participants also identified two economic fundamental goals: *low resource use in care setting* by preventing situations that require increased levels of staffing, equipment, and infrastructure, and *low direct costs of childbirth care* in monetary terms. These fundamental goals may gain significance in low-resource contexts, where it is essential to use resources consciously to provide the best possible care for everyone. Yet, the emphasis on resource utilization extends to middle-resource and high-resource contexts as well, as healthcare becomes increasingly economized. Higher body weight has indeed been linked to increased maternity care costs^51^ due to higher medical complexities and more frequent obstetric interventions.^68^ The expenses may burden healthcare systems and impose significant financial strain for women and their families. Given the increased prevalence of higher maternal body weight among women of lower socioeconomic status,^69 70^ the impact of these costs may be particularly detrimental. High out-of-pocket expenditures could impede access to care and affect biopsychosocial health outcomes. This study thus also draws attention to the pragmatic constraints faced by healthcare systems^71^ while striving to enhance both individual and population health for women with higher body weight.

The fundamental goals of childbirth care identified in our study may be broadly relevant to all women and their maternity care providers, regardless of body weight. However, balancing these multiple goals in the context of childbirth care for women with higher body weight can be especially complex. The goals may conflict, and may not be achieved simultaneously, which requires trade-offs to be made. As an initial exploration of potential goal prioritization among participants, we used direct rating of the perceived importance of the fundamental goals. In future studies, it will be essential to properly elicit stakeholder preferences regarding the achievement of these fundamental goals to guide decisions towards tailored, high-quality care.^20^ The trade-offs that stakeholders are willing to make will likely depend on their role in the decision, the decision situation, and the options being considered. Variations in goal achievement across options may strongly influence what is deemed to be the best possible care. Aligning decisions with the fundamental goals generated in a value-focused process therefore offers an opportunity to promote childbirth care that is person-centered, safe, sustainable, and responsive to the needs and preferences of women with higher body weight.

### Evaluation of the masterlist method to generate a diverse set of fundamental goals

Combining brainstorming with goal selection from the masterlist supported individual stakeholders in our healthcare context to generate a more diverse set of fundamental goals. This corresponds to the findings of Bond *et al*^26^ ^27^ and Haag *et al*^28^ in non-healthcare settings. While participants self-generated on average about 82% of their fundamental goals during individual brainstorming in our study, they recognized about 18% exclusively from the masterlist. Although less pronounced, this finding is in line with earlier research, which found that 25% and about 50% of the participants’ goals were solely generated with masterlist support.^26 28^ The less extreme result in our study may be explained by the well-established notion of high-quality childbirth care,^16^ making it more straightforward for women and their maternity care providers to think about goals than in other decision contexts. Yet, the additional use of the masterlist still resulted in a statistically significantly higher average number of fundamental goals compared with individual brainstorming alone, even in our sample of 21 participants. This suggests that the masterlist helped participants generate more diverse fundamental goals in our healthcare setting.

In our study, both self-generated and recognized fundamental goals were perceived similarly important. In contrast, self-generated goals from brainstorming were rated significantly more important than goals recognized from the masterlist in the study by Haag *et al.*^28^ Our result might indicate that the masterlist complemented personally relevant goals. Without the masterlist, about 15%–16% of the participants would not have generated the fundamental goals on biomedical safety that were perceived as most important overall (A1/A2; table 3; figure 2). This is again similar to previous research where 11% of the participants did not self-generate their most important goal.^28^ It is well-known in decision analysis that stakeholders often do not remember all goals during brainstorming that are fundamentally important to them, likely through an incomplete, myopic mental representation of the decision.^72^ A tendency to recall readily available concerns may lead to a narrow focus on salient goals in memory and thus to overlooking other goals.^26 72^ People are used to thinking in terms of possible options (‘alternative-focused thinking’), rather than underlying values (‘value-focused thinking’),^31^ and stakeholders in maternity care may typically assume that considered options are biomedically safe.^6 7^ Some participants may thus have focused on other potential benefits of such options during brainstorming, overlooking goals implicitly non-negotiable to them. Such goals have been referred to as taboo trade-offs, sacred, or protected values and relate to issues for which stakeholders are unwilling to make trade-offs.^73^,^75^ They may influence healthcare decision-making by limiting careful consideration of pertinent information.^75^ In this case, the masterlist would have supported the participants in explicitly articulating these values separately from the considered options.^73^

At the level of the entire sample, we found that a theoretical maximum of six participants would have been sufficient to capture the complete set of fundamental goals, and that no further fundamental goals were elicited from the additional 15 participants. Engaging more participants might thus not have resulted in a more diverse set of fundamental goals in our study, which aligns with earlier research.^28^ The finding suggests that the masterlist method may be especially valuable to identify care goals in busy healthcare settings, where recruiting stakeholders can be challenging. Implementing brainstorming followed by selecting goals from a masterlist could be relatively time efficient. However, complex healthcare topics may require involving more diverse stakeholders to generate a comprehensive set of fundamental care goals. An advantage of this method is therefore its applicability through paper-pencil questionnaires^26 27^ or online surveys,^28^ allowing for scalability and anonymized data collection if needed.^28^ This may be particularly helpful for collecting data from larger samples and for sensitive topics such as body weight.

In summary, our methodological results indicate that combining individual brainstorming with goal selection from a masterlist is an easy-to-use and useful method for identifying diverse healthcare goals, requiring only a limited number of participants.

### Strengths, limitations, and research needs

To our knowledge, this was the first study to identify a diverse set of fundamental goals of childbirth care for women with higher body weight involving multiple key stakeholders and drawing on an established goal-elicitation method from decision analysis. While we do not to claim that the identified fundamental goals are universal or equally important to all women with higher body weight and their maternity care providers, the study does provide a framework for elucidating potential childbirth care goals and their underlying values. This framework may inform decision-making processes and further research in the field. Additionally, we have demonstrated the masterlist’s value in prompting the articulation of otherwise overlooked goals in a healthcare context.

However, the study sample was limited to 21 white, educated, female care recipients and providers in the high-resource Swiss maternity care setting. It does not reflect the diversity of people^76^ involved in childbirth care decisions. Future studies could validate and potentially expand the provided framework of fundamental goals to include diverse socioeconomic and cultural contexts, different healthcare systems, and populations, such as the general birthing population or women with other complexities. This would allow for encompassing goals that may not have been captured within the specific context of Swiss maternity care for women with higher body weight. Moreover, it could be valuable for assessing the transferability of our findings.

To advance childbirth care for women with higher body weight, ongoing research is needed to evaluate the utility of the fundamental goals using methods such as Multi-Criteria Decision Analysis (MCDA).^20 21^ This further involves defining a diverse set of potential options for childbirth care, assessing how well each option achieves the fundamental goals with scientific methods, and eliciting stakeholder preferences for making trade-offs between achieving these multidimensional goals.^20^ Aggregating all this information into a comprehensive MCDA model will allow to evaluate the overall value of different childbirth care options and understand how stakeholder preferences may impact the provision of appropriate care. In this study, we took initial steps towards structuring the decision based on stakeholders’ values. Combining these insights with an MCDA process could establish a sound and scientific basis for informing decision-making.

## Conclusions

This study offers valuable insights into identifying fundamental goals of childbirth care for women with higher body weight. By providing empirical evidence on multidimensional goals, it establishes a framework for facilitating decision-making on childbirth care. Moreover, the set of fundamental goals may serve as a benchmark for evaluating and improving childbirth services. Our approach thereby contributes to a better understanding and promotes discourse about what is fundamentally valued from the perspectives of multiple key stakeholders. Advancing childbirth care for women with higher body weight requires ongoing research, inclusivity, and a commitment to person-centered and evidence-based healthcare.

In addition, the study suggests that individual brainstorming combined with goal selection from a pre-established masterlist may be a useful method for generating diverse fundamental goals in a healthcare context, even with relatively few participants. This method has the potential to guide future efforts in identifying the goals that stakeholders consider fundamentally important to achieve during healthcare episodes.

## Supplementary material

10.1136/bmjopen-2024-086409 online supplemental file 1

## Data Availability

All data relevant to the study are included in the article or uploaded as supplementary information.
