# Supplementary material for: Identifying fundamental goals of childbirth care for women with higher body weight in Swiss maternity care: an embedded mixed methods multi-stakeholder study
Source: BMJ Open. 2025 Jul 22;15(7):e086409. doi: 10.1136/bmjopen-2024-086409 (PMC12306249; doi:10.1136/bmjopen-2024-086409)
Supplement: online supplemental file 1 [file bmjopen-15-7-s001.pdf]

Identifying fundamental goals of childbirth care for women with higher body weight in Swiss maternity care: an embedded mixed methods multi-stakeholder study

Supplemental material

Authors

Carmen Wyss[1,2]; Jennifer Inauen[3]; Judit Lienert[4]; Evelyne M. Aubry[1]

Affiliations

[1] Applied Research and Development, Division of Midwifery, Department of Health Professions, Bern University of Applied Sciences, Switzerland

[2] Graduate School for Health Sciences, University of Bern, Switzerland

[3] Department of Health Psychology and Behavioral Medicine, Institute of Psychology, University of Bern, Switzerland

[4] Decision Analysis Group, Department of Environmental Social Sciences, Swiss Federal Institute of Aquatic Science and Technology (Eawag), Dübendorf, Switzerland

Address information for correspondence

Carmen Wyss, Applied Research and Development, Division of Midwifery, Department of Health Professions, Bern University of Applied Sciences, Murtenstrasse 10, 3008 Bern, Switzerland.

E-mail: carmen.wyss@bfh.ch, phone: +41 31 848 34 99.

Contents

S1. Masterlist of childbirth care goals 2

S2. Workshop and interview guide 5

S3. Thematic analysis 11

S4. Means-ends network 12

References 13

S1. Masterlist of childbirth care goals

Goal	Informed by
Low maternal morbidity	NICE quality standard. *Intrapartum care*. 2015, updated 2017. https://www.nice.org.uk/guidance/qs105 [1]<br>NICE quality standard. *Intrapartum care: existing medical conditions and obstetric complications*. 2020. https://www.nice.org.uk/guidance/qs192 [2]<br>International Consortium for Health Outcome Measurement [ICHOM]. *Pregnancy & childbirth data collection reference guide*. 2017. https://ichom.org/files/medical-conditions/pregnancy-and-childbirth/pregnancy-childbirth-reference-guide.pdf [3]<br>Dadouch R, Faheim M, Susini O, Sedra S, Showell M, D'Souza R. Variation in outcome reporting in studies on obesity in pregnancy: a systematic review. *Clin Obes*. 2019;9(6):e12341. doi:10.1111/cob.12341 [4]<br>Dadouch R, Hall C, Du Mont J, D'Souza R. Obesity in pregnancy - Patient-reported outcomes in qualitative research: a systematic review. *J Obstet Gynaecol Can*. 2020;42(8):1001-1011. doi:10.1016/j.jogc.2019.09.011 [5]
Low maternal mortality	NICE quality standard. *Intrapartum care*. 2015, updated 2017. https://www.nice.org.uk/guidance/qs105 [1]<br>NICE quality standard. *Intrapartum care: existing medical conditions and obstetric complications*. 2020. https://www.nice.org.uk/guidance/qs192 [2]<br>International Consortium for Health Outcome Measurement [ICHOM]. *Pregnancy & childbirth data collection reference guide*. 2017. https://ichom.org/files/medical-conditions/pregnancy-and-childbirth/pregnancy-childbirth-reference-guide.pdf [3]<br>Dadouch R, Hall C, Du Mont J, D'Souza R. Obesity in pregnancy - Patient-reported outcomes in qualitative research: a systematic review. *J Obstet Gynaecol Can*. 2020;42(8):1001-1011. doi:10.1016/j.jogc.2019.09.011 [5]
Low neonatal morbidity	NICE quality standard. *Intrapartum care*. 2015, updated 2017. https://www.nice.org.uk/guidance/qs105 [1]<br>NICE quality standard. *Intrapartum care: existing medical conditions and obstetric complications*. 2020. https://www.nice.org.uk/guidance/qs192 [2]<br>Downe S, Finlayson K, Oladapo OT, Bonet M, Gulmezoglu AM. What matters to women during childbirth: a systematic qualitative review. *PloS One*. 2018;13(4):e0194906. doi:10.1371/journal.pone.0194906 [6]<br>International Consortium for Health Outcome Measurement [ICHOM]. *Pregnancy & childbirth data collection reference guide*. 2017. https://ichom.org/files/medical-conditions/pregnancy-and-childbirth/pregnancy-childbirth-reference-guide.pdf [3]<br>Dadouch R, Faheim M, Susini O, Sedra S, Showell M, D'Souza R. Variation in outcome reporting in studies on obesity in pregnancy: a systematic review. *Clin Obes*. 2019;9(6):e12341. doi:10.1111/cob.12341 [4]<br>Dadouch R, Hall C, Du Mont J, D'Souza R. Obesity in pregnancy - Patient-reported outcomes in qualitative research: a systematic review. *J Obstet Gynaecol Can*. 2020;42(8):1001-1011. doi:10.1016/j.jogc.2019.09.011 [5]
Low neonatal mortality	NICE quality standard. *Intrapartum care*. 2015, updated 2017. https://www.nice.org.uk/guidance/qs105 [1]<br>NICE quality standard. *Intrapartum care: existing medical conditions and obstetric complications*. 2020. https://www.nice.org.uk/guidance/qs192 [2]<br>Downe S, Finlayson K, Oladapo OT, Bonet M, Gulmezoglu AM. What matters to women during childbirth: a systematic qualitative review. *PloS One*. 2018;13(4):e0194906. doi:10.1371/journal.pone.0194906 [6]<br>International Consortium for Health Outcome Measurement [ICHOM]. *Pregnancy & childbirth data collection reference guide*. 2017. https://ichom.org/files/medical-conditions/pregnancy-and-childbirth/pregnancy-childbirth-reference-guide.pdf [3]<br>Dadouch R, Faheim M, Susini O, Sedra S, Showell M, D'Souza R. Variation in outcome reporting in studies on obesity in pregnancy: a systematic review. *Clin Obes*. 2019;9(6):e12341. doi:10.1111/cob.12341 [4]<br>Dadouch R, Hall C, Du Mont J, D'Souza R. Obesity in pregnancy - Patient-reported outcomes in qualitative research: a systematic review. *J Obstet Gynaecol Can*. 2020;42(8):1001-1011. doi:10.1016/j.jogc.2019.09.011 [5]
Undisturbed physiological labor processes (No obstetric interventions)	Downe S, Finlayson K, Oladapo OT, Bonet M, Gulmezoglu AM. What matters to women during childbirth: a systematic qualitative review. *PloS One*. 2018;13(4):e0194906. doi:10.1371/journal.pone.0194906 [6]<br>Saturno-Hernández PJ, Martínez-Nicolás I, Moreno-Zegbe E, Fernández-Elorriaga M, Poblano-Verástegui O. Indicators for monitoring maternal and neonatal quality care: a systematic review. *BMC Pregnancy Childbirth*. 2019;19(1):25. doi:10.1186/s12884-019-2173-2 [7]

Good mother-child-attachment (Bonding)	International Consortium for Health Outcome Measurement [ICHOM]. *Pregnancy & childbirth data collection reference guide*. 2017. https://ichom.org/files/medical-conditions/pregnancy-and-childbirth/pregnancy-childbirth-reference-guide.pdf [3]<br>Smith V, Daly D, Lundgren I, Eri T, Benstoem C, Devane D. Salutogenically focused outcomes in systematic reviews of intrapartum interventions: a systematic review of systematic reviews. *Midwifery*. 2014;30(4):e151-6. doi:10.1016/j.midw.2013.11.002 [8]<br>Korst LM, Fridman M, Saeb S, Greene N, Fink A, Gregory KD. The development of a conceptual framework and preliminary item bank for childbirth-specific patient-reported outcome measures. *Health Serv Res*. 2018;53(5):3373-3399. doi:10.1111/1475-6773.12856 [9]
High satisfaction with care	NICE quality standard. *Intrapartum care*. 2015, updated 2017. https://www.nice.org.uk/guidance/qs105 [1]<br>NICE quality standard. *Intrapartum care: existing medical conditions and obstetric complications*. 2020. https://www.nice.org.uk/guidance/qs192 [2]<br>World Health Organization [WHO]. *Standards for improving quality of maternal and newborn care in health facilities*. 2016. https://www.who.int/publications/i/item/9789241511216 [10]<br>World Health Organization [WHO]. *WHO recommendations: intrapartum care for a positive childbirth experience*. 2018. https://www.who.int/publications/i/item/9789241550215 [11]<br>International Consortium for Health Outcome Measurement [ICHOM]. *Pregnancy & childbirth data collection reference guide*. 2017. https://ichom.org/files/medical-conditions/pregnancy-and-childbirth/pregnancy-childbirth-reference-guide.pdf [3]<br>Smith V, Daly D, Lundgren I, Eri T, Benstoem C, Devane D. Salutogenically focused outcomes in systematic reviews of intrapartum interventions: a systematic review of systematic reviews. *Midwifery*. 2014;30(4):e151-6. doi:10.1016/j.midw.2013.11.002 [8]<br>Dadouch R, Faheim M, Susini O, Sedra S, Showell M, D'Souza R. Variation in outcome reporting in studies on obesity in pregnancy: a systematic review. *Clin Obes*. 2019;9(6):e12341. doi:10.1111/cob.12341 [4]
Positive childbirth experience	World Health Organization [WHO]. *WHO recommendations: intrapartum care for a positive childbirth experience*. 2018. https://www.who.int/publications/i/item/9789241550215 [11]<br>World Health Organization [WHO]. *Standards for improving quality of maternal and newborn care in health facilities*. 2016. https://www.who.int/publications/i/item/9789241511216 [10]<br>NICE quality standard. *Intrapartum care*. 2015, updated 2017. https://www.nice.org.uk/guidance/qs105 [1]<br>Downe S, Finlayson K, Oladapo OT, Bonet M, Gulmezoglu AM. What matters to women during childbirth: a systematic qualitative review. *PloS One*. 2018;13(4):e0194906. doi:10.1371/journal.pone.0194906 [6]<br>International Consortium for Health Outcome Measurement [ICHOM]. *Pregnancy & childbirth data collection reference guide*. 2017. https://ichom.org/files/medical-conditions/pregnancy-and-childbirth/pregnancy-childbirth-reference-guide.pdf [3]<br>Smith V, Daly D, Lundgren I, Eri T, Benstoem C, Devane D. Salutogenically focused outcomes in systematic reviews of intrapartum interventions: a systematic review of systematic reviews. *Midwifery*. 2014;30(4):e151-6. doi:10.1016/j.midw.2013.11.002 [8]<br>Dadouch R, Hall C, Du Mont J, D'Souza R. Obesity in pregnancy - Patient-reported outcomes in qualitative research: a systematic review. *J Obstet Gynaecol Can*. 2020;42(8):1001-1011. doi:10.1016/j.jogc.2019.09.011 [5]
Safe care environment	NICE quality standard. *Intrapartum care*. 2015, updated 2017. https://www.nice.org.uk/guidance/qs105 [1]<br>Downe S, Finlayson K, Oladapo OT, Bonet M, Gulmezoglu AM. What matters to women during childbirth: a systematic qualitative review. *PloS One*. 2018;13(4):e0194906. doi:10.1371/journal.pone.0194906 [6]
Well-informed woman	World Health Organization [WHO]. *Standards for improving quality of maternal and newborn care in health facilities*. 2016. https://www.who.int/publications/i/item/9789241511216 [10]<br>World Health Organization [WHO]. *WHO recommendations: intrapartum care for a positive childbirth experience*. 2018. https://www.who.int/publications/i/item/9789241550215 [11]
Involvement of woman in decision	World Health Organization [WHO]. *Standards for improving quality of maternal and newborn care in health facilities*. 2016. https://www.who.int/publications/i/item/9789241511216 [10]<br>NICE quality standard. *Intrapartum care: existing medical conditions and obstetric complications*. 2020. https://www.nice.org.uk/guidance/qs192 [2]<br>World Health Organization [WHO]. *WHO recommendations: intrapartum care for a positive childbirth experience*. 2018. https://www.who.int/publications/i/item/9789241550215 [11]<br>Dadouch R, Hall C, Du Mont J, D'Souza R. Obesity in pregnancy - Patient-reported outcomes in qualitative research: a systematic review. *J Obstet Gynaecol Can*. 2020;42(8):1001-1011. doi:10.1016/j.jogc.2019.09.011 [5]

Dignity	World Health Organization [WHO]. *Standards for improving quality of maternal and newborn care in health facilities*. 2016. https://www.who.int/publications/i/item/9789241511216 [10]<br>World Health Organization [WHO]. *WHO recommendations: intrapartum care for a positive childbirth experience*. 2018. https://www.who.int/publications/i/item/9789241550215 [11]
Confidentiality	World Health Organization [WHO]. *Standards for improving quality of maternal and newborn care in health facilities*. 2016. https://www.who.int/publications/i/item/9789241511216 [10]<br>World Health Organization [WHO]. *WHO recommendations: intrapartum care for a positive childbirth experience*. 2018. https://www.who.int/publications/i/item/9789241550215 [11]
Privacy	World Health Organization [WHO]. *Standards for improving quality of maternal and newborn care in health facilities*. 2016. https://www.who.int/publications/i/item/9789241511216 [10]<br>World Health Organization [WHO]. *WHO recommendations: intrapartum care for a positive childbirth experience*. 2018. https://www.who.int/publications/i/item/9789241550215 [11]<br>Dadouch R, Hall C, Du Mont J, D'Souza R. Obesity in pregnancy - Patient-reported outcomes in qualitative research: a systematic review. *J Obstet Gynaecol Can*. 2020;42(8):1001-1011. doi:10.1016/j.jogc.2019.09.011 [5]
No stigmatization and discrimination	World Health Organization [WHO]. *Standards for improving quality of maternal and newborn care in health facilities*. 2016. https://www.who.int/publications/i/item/9789241511216 [10]<br>Dadouch R, Hall C, Du Mont J, D'Souza R. Obesity in pregnancy - Patient-reported outcomes in qualitative research: a systematic review. *J Obstet Gynaecol Can*. 2020;42(8):1001-1011. doi:10.1016/j.jogc.2019.09.011 [5]
Low need of staffing	Expert knowledge<br>Saturno-Hernández PJ, Martínez-Nicolás I, Moreno-Zegbe E, Fernández-Elorriaga M, Poblano-Verástegui O. Indicators for monitoring maternal and neonatal quality care: a systematic review. *BMC Pregnancy Childbirth*. 2019;19(1):25. doi:10.1186/s12884-019-2173-2 [7]
Low need of equipment and infrastructure	Dadouch R, Faheim M, Susini O, Sedra S, Showell M, D'Souza R. Variation in outcome reporting in studies on obesity in pregnancy: a systematic review. *Clin Obes*. 2019;9(6):e12341. doi:10.1111/cob.12341 [4]<br>Dadouch R, Hall C, Du Mont J, D'Souza R. Obesity in pregnancy - Patient-reported outcomes in qualitative research: a systematic review. *J Obstet Gynaecol Can*. 2020;42(8):1001-1011. doi:10.1016/j.jogc.2019.09.011 [5]<br>Saturno-Hernández PJ, Martínez-Nicolás I, Moreno-Zegbe E, Fernández-Elorriaga M, Poblano-Verástegui O. Indicators for monitoring maternal and neonatal quality care: a systematic review. *BMC Pregnancy Childbirth*. 2019;19(1):25. doi:10.1186/s12884-019-2173-2 [7]<br>Expert knowledge
Low costs	Dadouch R, Faheim M, Susini O, Sedra S, Showell M, D'Souza R. Variation in outcome reporting in studies on obesity in pregnancy: a systematic review. *Clin Obes*. 2019;9(6):e12341. doi:10.1111/cob.12341 [4]<br>Dadouch R, Hall C, Du Mont J, D'Souza R. Obesity in pregnancy - Patient-reported outcomes in qualitative research: a systematic review. *J Obstet Gynaecol Can*. 2020;42(8):1001-1011. doi:10.1016/j.jogc.2019.09.011 [5]<br>Expert knowledge

S2. Workshop and interview guide

Important notes:

* The subsequent guide constitutes an abriged English translation of the original German version.
* Based in parts on the interview guide «Gesprächsleitfaden für Akteure und Projektpartner» by Beutler et al. (2024).[12]
* Modified Nominal Group Technique based on Manera et al. (2019), Keller (2019), Van de Ven and Delbecq (1972), and Delbecq and Van de Ven (1971).[13-16]
* Masterlist support for generating a diverse set of goals based on Bond et al. (2008), Bond et al. (2010), and Haag et al. (2019).[17-19]
* Masterlist and literature sources are provided in supplemental material S1.
* Foundations on goals and goal development in structured decision-making from Gregory et al. (2012).[20]

Part A: Introduction and formalities

Welcome

- Greet the participant(s) and express appreciation for their time and effort to participate.

Workshop/interview language

- Ask if anyone prefers the workshop to be conducted in standard German instead of Swiss German (group workshops).
- Inquire about the participant's preferred language for the interview: Swiss German, standard German, or English (individual interviews).

Form of address

- Ask the participant(s) how they prefer to be addressed (formal or informal).

Introduction

- Introduce the facilitator(s) of the workshop/interview.
- Ask if the participant(s) would like to briefly introduce themselves.

Sensitive terminology

- Explain that the focus of the workshop/interview will be on childbirth care for women with higher body weight (BMI ≥ 30 kg/m^2).
- Mention that the term "obesity" is commonly used by medical professionals, but acknowledge that body weight can be a sensitive topic.
- Ask the participant(s) about their preferences for terminology/language related to body weight.
- Clarify that language preferences may vary between individuals. If a term is inadvertently used that does not align with their needs, they are encouraged to let you know.

Study's topic and rationale

- Explain that the research focuses on <u>childbirth care for women with higher body weight</u> (BMI ≥ 30 kg/m^2).
- Highlight that all women should receive high-quality care during childbirth.
- Briefly mention that research suggests that childbirth care for women with higher body weight may not always be optimal.
- Emphasize the importance of identifying what needs to be achieved to provide optimal care in order to improve childbirth care services.

Purpose of the workshop or interview

- Clarify that the study aims to explore what consitutes optimal care.
- Explain that the focus is on identifying *goals*, i.e., valued "outcomes", "results" or "conditions" that childbirth care should achieve.
- Encourage the participant(s) to share their perspectives on what may be fundamentally valued.
- Mention that the purpose of the interview is not about *how* to achieve the goals, i.e., not about options.
- Inform the participant(s) that the study gathers insights from both women and maternity care providers.
- Let them know that once the goals are identified, the next study will explore how best to achieve them.

Data collection structure and process

- Explain that data collection will be structured and systematically moderated, beginning with prepared tasks.
- Outline the process:
  1. Individual brainstorming
  2. Comparison with a pre-established masterlist
  3. Discussion/elaboration of the goals

Open participation

- Reassure the participant(s) that there are no right or wrong answers.
- Emphasize that their perspectives and inputs are valued without judgment.
- Emphasize the importance of allowing space for all opinions, even if they differ from personal views. The study aims to capture diverse perspectives, not to reach consensus (group workshops).
- Encourage the participant(s) to ask questions, seek clarification, or pause the workshop/interview if needed.

Questions

- Ask if there are any questions or uncertainties before proceeding.

Data protection and consent

- Acknowledge that the participant(s) has/have signed the consent form before the workshop/interview.
- Explain that recording of the workshop/interview is essential for scientific evaluation and helps ensure accuracy.
- Ask for explicit permission to record the workshop/interview (video recording for online sessions; audio recording for face-to-face sessions).
- If the participant(s) agree(s), start the recording.
- Begin the recording with a formal statement including the session number, date, and confidentiality assurances:
  - The recording will be handled confidentially.
  - All research team members are bound to confidentiality.
  - Any data used for research or publications will be fully anonymized to prevent identification.

Part B: Goal elicitation

Phase 1: Individual brainstorming

1st Round

- Display the question: What goals should be achieved through childbirth care for women with higher body weight?
- Instruct the participant(s) to write down all ideas that come to mind.
- Remind them that there are no right or wrong answers.
- Allow enough time for the first brainstorming round.
- Acknowledge the efforts of the participant(s).

2nd Round

- Encourage the participant(s) to consider additional perspectives beyond their own and challenge them to double the number of goals they list.
- Allow enough time for the second brainstorming round.
- Acknowledge the efforts of the participant(s).

Differentiating goals from options

- Explain that when asked about *what* should be achieved, people often mention *how* things can be achieved (options).
- Ask the participant(s) to check if they wrote down any options and, if so, what goals they want to achieve with these options.

Phase 2: Completion of goals using the masterlist

- Introduce the masterlist to complement the goals that were generated during brainstorming.

- Clarify that the masterlist contains possible goals for childbirth care. These goals may or may not align with personal opinions, and some may be more relevant to certain people than for others.

Goal comparison

- Ask the participant(s) to identify the goals that appear both on their personal list and the masterlist.
- Record the goals that appear on both lists for each participant. These goals are defined as *self-generated* during individual brainstorming as they first appeared on the personal list of the participant(s).

Additional recognized goals from the masterlist

- Ask the participant(s) to identify the goals that appear only on the masterlist but seem relevant to them.
- Record the selected new goals from the masterlist for each participant. These goals are defined as *recognized* from the masterlist.

Additional self-generated goals

- Ask the participant(s) to identify the goals that are only on their personal list and not on the masterlist.
- Record the goals that appear only on the personal list for each participant. These goals are defined as *self-generated* during individual brainstorming.

- Thank the participant(s) for comparing their personal list with the masterlist and for complementing it with additional new goals from the masterlist.

Phase 3: Discussion/elaboration of the goals

- Explore the goals that have been self-generated or recognized.
- Record any new goals that may emerge during the workshop/interview.
- Display all goals virtually or on paper for the participant(s) to confirm or revise as needed.
- Adjust the wording of goals if necessary.
- If no further goals are mentioned, ask specifically about unaddressed goals.
- If time is limited, move discussion/elaboration forward while ensuring all goals are considered.

New goals

- Ask the participant(s) to explain the relevance of their goals.
- Encourage the participant(s) to engage with each other if they share similar goals (group workshops).
- Inquire if any goals on their personal list(s) are similar but not identical to the masterlist. Explore the differences and why they are considered important.
- Ask which goals seem particularly relevant when making decisions about childbirth care for women with higher body weight and why.
- Ask the participant(s) to categorize goals.

Emphasize broad input

- Encourage the participant(s) to mention any goals they may have listed, even those that may not apply to the them but could be relevant to others.
- Clarify that all perspectives are welcome.
- Acknowledge that disagreements may arise and encourage open sharing without the need for consensus (group workshops).

Body weight consideration

- Inquire as to whether the participant(s) consider(s) the goals of childbirth care to be dependent on body weight.

Final review of goals

- Provide a clear and concise summary of all goals and their relevance.
- Ask if there are any additional goals that have not yet been shared.
- Check with the participant(s) if anything important was overlooked or needs further consideration.
- Review the categorization of goals with the participant(s) and ask if any adjustments are needed.
- Ensure all goals are clear and understood.

Possible facilitator prompts throughout the discussion/elaboration

Encouraging responses

- Why is this goal important?
- What do you hope to achieve?
- Why do you want to achieve this?
- How would you describe this goal in more detail?
- Can you provide further clarification on this goal?
- Can you provide an example?

Organizing and categorizing goals

- How does this goal relate to others?
- Are there goals that are similar?
- Are there goals that influence each other?
- What is the difference between … and …?

Goal hierarchy

- When would you consider this goal achieved?
- How could this goal be measured?
- What overarching goal would you asign this aspect to?
- Why do you want to achieve this specific goal?

Distinguishing goals from care options

- It seems like this stament is more about a care option – something we can ‘do’ to achieve a goal. What are you hoping to achieve with this option? Why is it important?

Possible questions to refine goals[cf.20]

- Completeness: Are any important goals missing? Have all key concerns been addressed?
- Conciseness: Are any goals unnecessary or ambiguous?
- Understandability: Are the goals clear and understandable? Are there any terms that need clarification?
- Feasibility: Can the goal realistically be influenced by childbirth care?
- Non-redundancy: Are these goal distinct? Overlapping? Do they express similar aspects of the same goal?
- Measurability: How can this goal be quantified? What specific criteria must be met to consider it achieved?
- Preferential independence: Are there goals that are dependent on each other? Can their importance only be judged in relation to others?

Part 3: Closing

Final reflection

- Offer the participant(s) the opportunity to share their thoughts on the workshop/interview and to ask questions.
- Acknowledge their feedback as a means to improve future workshops/interviews.

Follow-up

- Inform the participant(s) that they will receive an email with a link to a short questionnaire to rate the identified goals and to provide some demographic information.
- Assure them that all data will be treated confidentially.

Thanks and acknowledgement

- Thank the participant(s) for their time and valuable contributions.
- Emphasize the importance of their perspective for the study.

Contact and support

- Encourage the participant(s) to reach out anytime with questions or concerns related to the workshop/interview.

Goodbye

- Express gratitude, wish participants well, and say goodbye.

S3. Thematic analysis

Unique goals	Number of goals merged[a]	Category	Subtheme	Theme
Low maternal morbidity*	1	Prevent maternal biomedical health risks		Ensure biomedical safety
Low maternal mortality*	1			
Vital maternal state	1			
Prevention of maternal complications	1			
Prevention of maternal accidents	1			
Low neonatal morbidity*	1	Prevent neonatal biomedical health risks		
Low neonatal mortality*	1			
Vital neonatal state	1			
Undisturbed physiological labor processes*	1	Focus on labor and childbirth physiology	Empower physiological labor and childbirth processes	Empower physiological processes
Physiological childbirth process	1			
Physiological vaginal birth	3			
Focus on physiology	3			
Avoidance of unnecessary cesarean section	1	Avoid unnecessary interventions		
No unnecessary interventions on the woman	3			
No unnecessary interventions on the newborn	3		Support positive initiation of bonding and breastfeeding	
Immediate bonding	1	Facilitate parent-child-attachment		
Good mother-child-attachment*	1			
Good father/partner-child-attachment	2			
Successful/positive breastfeeding initiation	4	Initiate successful breastfeeding		
High satisfaction with care*	1	High satisfaction with care		Facilitate a positive psychosocial experience of care
Positive childbirth experience*	1	Positive childbirth experience		
Safe care environment*	1	Ensure a safe care environment	Create a safe and supporting setting	
Protected physical integrity	1			
Protected psychological integrity	1			
Prevention of (re)traumatization	1			
Known place and people	4	Build good rapport and level of trust		
Good rapport between woman and care providers	2			
Mutual candor	1			
Physical and emotional caring	3	Provide adequate physical and emotional support		
Ease and comfort during labor and childbirth	2			
Harmonious ambiance	2			
Orientation	1			
Involvement of childbirth companion	3	Involve the childbirth companion appropriately		
Focus on the woman's individuality	9	Valorize the individual woman	Make care person-centered	
Taking the woman seriously	6			
Responsiveness to needs	1	Respond to the needs of the woman		
Well-informed woman*	1	Empower the woman to make decisions		
Involvement of woman in decisions*	1			
Shared decision-making	1			
Sense of self-responsibility	2			
Self-determined childbirth	6	Promote the woman's self-determination		
Power of decision with parents	1			
Realistic situational assessment	3	Give realistic and honest information	Communicate constructively	
Honest communication by care providers	2			
Sensitive communication by care providers	1	Use sensitive and balanced approach to discuss implications		
Positive anticipation of childbirth by balanced communication	3			
Dignity*	1	Preserve the woman's dignity	Treat each woman with respect	
Confidentiality*	1	Respect confidentiality		
Privacy*	1	Respect privacy		
No stigmatization and discrimination*	1	Care for women without prejudice, stigmatization, and discrimination		
No sole focus on body weight	5			
Unprejudiced care	4			
No dogmata in care	1			
Discrimination-sensitivity	1			
Respectful communication	3	Use unbiased and respectful language		
Low physical strain for care providers	2	Reduce physical strain for care providers		Use resources consciously
Low need of staffing*	1	Consider availability of resources in settings		
Low need of equipment and infrastructure*	1			
Low costs *	1	Consider direct monetary costs		

Note: [a] Merging of goals sharing the same underlying meaning across all data collection events to prevent redundancy (cf. Table 1, step 6). * On the masterlist (cf. supplemental material S1)

S4. Means-ends network

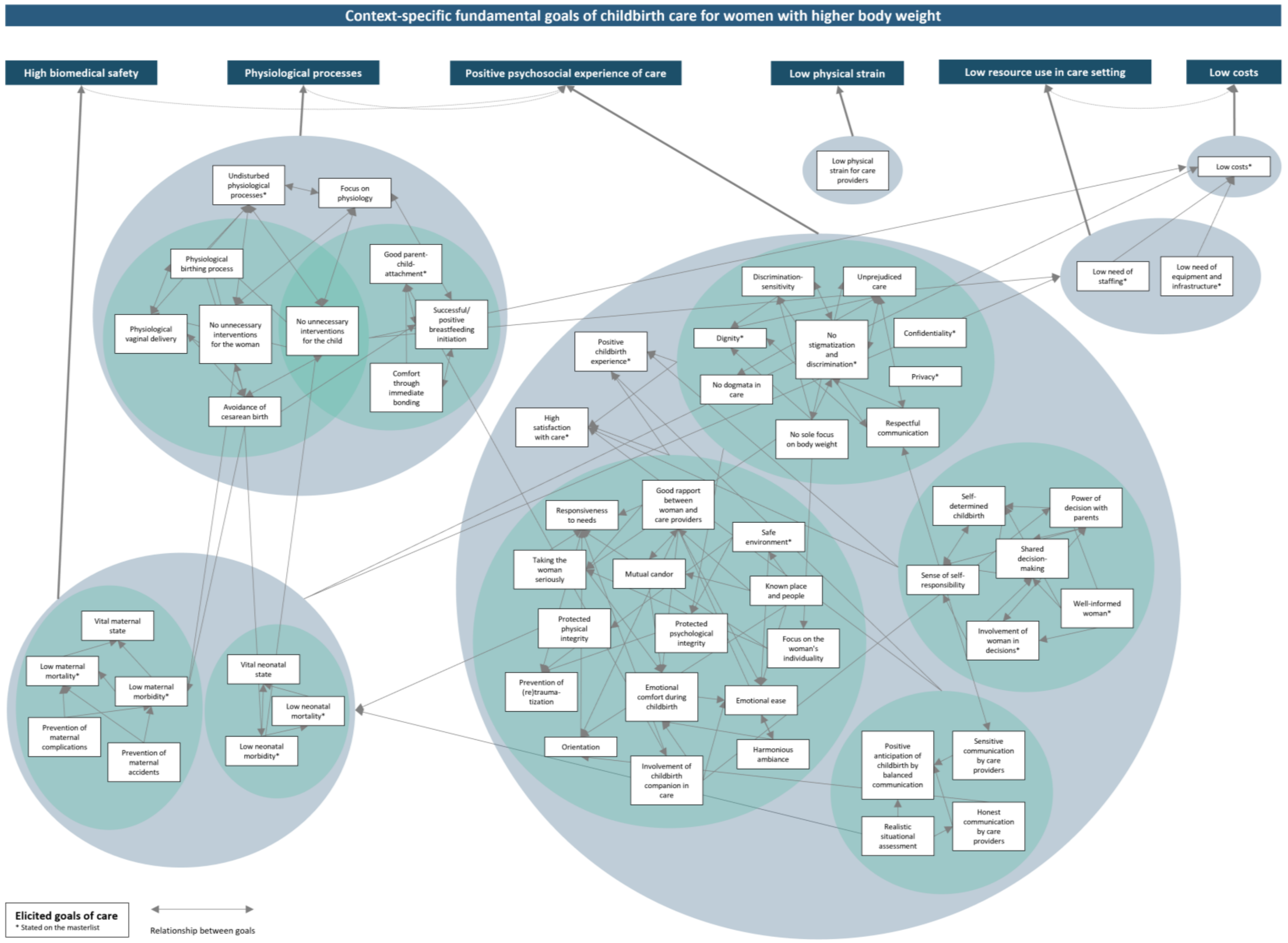

References

1. NICE quality standard. *Intrapartum care*. 2015, updated 2017. https://www.nice.org.uk/guidance/qs105
2. NICE quality standard. *Intrapartum care: existing medical conditions and obstetric complications*. 2020. https://www.nice.org.uk/guidance/qs192
3. International Consortium for Health Outcome Measurement [ICHOM]. *Pregnancy & childbirth data collection reference guide*. 2017. https://ichom.org/files/medical-conditions/pregnancy-and-childbirth/pregnancy-childbirth-reference-guide.pdf
4. Dadouch R, Faheim M, Susini O, Sedra S, Showell M, D'Souza R. Variation in outcome reporting in studies on obesity in pregnancy: a systematic review. *Clin Obes*. 2019;9(6):e12341. doi:10.1111/cob.12341
5. Dadouch R, Hall C, Du Mont J, D'Souza R. Obesity in pregnancy - Patient-reported outcomes in qualitative research: a systematic review. *J Obstet Gynaecol Can*. 2020;42(8):1001-1011. doi:10.1016/j.jogc.2019.09.011
6. Downe S, Finlayson K, Oladapo OT, Bonet M, Gulmezoglu AM. What matters to women during childbirth: a systematic qualitative review. *PloS one*. 2018;13(4):e0194906. doi:10.1371/journal.pone.0194906
7. Saturno-Hernández PJ, Martínez-Nicolás I, Moreno-Zegbe E, Fernández-Elorriaga M, Poblano-Verástegui O. Indicators for monitoring maternal and neonatal quality care: a systematic review. *BMC Pregnancy Childbirth*. 2019;19(1):25. doi:10.1186/s12884-019-2173-2
8. Smith V, Daly D, Lundgren I, Eri T, Benstoem C, Devane D. Salutogenically focused outcomes in systematic reviews of intrapartum interventions: a systematic review of systematic reviews. *Midwifery*. 2014;30(4):e151-6. doi:10.1016/j.midw.2013.11.002
9. Korst LM, Fridman M, Saeb S, Greene N, Fink A, Gregory KD. The development of a conceptual framework and preliminary item bank for childbirth-specific patient-reported outcome measures. *Health Serv Res*. 2018;53(5):3373-3399. doi:10.1111/1475-6773.12856
10. World Health Organization [WHO]. *Standards for improving quality of maternal and newborn care in health facilities*. 2016. https://www.who.int/publications/i/item/9789241511216
11. World Health Organization [WHO]. *WHO recommendations: intrapartum care for a positive childbirth experience*. 2018. https://www.who.int/publications/i/item/9789241550215
12. Beutler P, Larsen TA, Maurer M, Staufer P, Lienert J. A participatory Multi-Criteria Decision Analysis framework reveals transition potential towards non-grid wastewater management. *J Environ Manage*. 2024;367. doi:10.1016/j.jenvman.2024.121962
13. Manera K, Hanson CS, Gutman T, Tong A. Consensus methods: nominal group technique. In: Liamputtong P, ed. *Handbook of Research Methods in Health Social Sciences*. Springer; 2019.
14. Keller S. *Nominal Groups*. 2019. https://sswm.info/planning-and-programming/decision-making/deciding-community/nominal-groups
15. Van de Ven AH, Delbecq AL. The nominal group as a research instrument for exploratory health studies. *Am J Public Health*. Mar 1972;62(3):337-42. doi:10.2105/ajph.62.3.337

16. Delbecq AL, Van de Ven AH. A group process model for problem identification and program planning. *J Appl Behav Sci*. 1971;7(4):466-492. doi:10.1177/002188637100700404
17. Bond SD, Carlson KA, Keeney RL. Generating objectives: can decision makers articulate what they want? *Manag Sci*. 2008;54(1):56-70.
18. Bond SD, Carlson KA, Keeney RL. Improving the generation of decision objectives. *Decis Anal*. 2010;7(3):238-255. doi:10.1287/deca.1100.0172
19. Haag F, Zürcher S, Lienert J. Enhancing the elicitation of diverse decision objectives for public planning. *Eur J Oper Res*. 2019;279(3):912-928. doi:10.1016/j.ejor.2019.06.002
20. Gregory R, Failing L, Harstone M, Long G, McDaniels T, Ohlson D. *Structured decision making: a practical guide to environmental management choices*. Wiley-Blackwell; 2012.
