# Supplementary material for: Identifying fundamental goals of childbirth care for women with higher body weight in Swiss maternity care: an embedded mixed methods multi-stakeholder study
Source: BMJ Open. 2025 Jul 22;15(7):e086409. doi: 10.1136/bmjopen-2024-086409 (PMC12306249; doi:10.1136/bmjopen-2024-086409)
Supplement: Supplementary file 2 [file bmjopen-2024-086409.reviewer_comments.pdf]

PEER REVIEW HISTORY

BMJ Open publishes all reviews undertaken for accepted manuscripts. Reviewers are asked to complete a checklist review form and are provided with free text boxes to elaborate on their assessment. These free text comments are reproduced below.

ARTICLE DETAILS

Title (Provisional)

Identifying fundamental goals of childbirth care for women with higher body weight in Swiss maternity care: An exploratory multi-stakeholder approach

Authors

Wyss, Carmen; Inauen, Jennifer; Lienert, Judit; Aubry, Evelyne M.

VERSION 1 - REVIEW

**Reviewer**	**1**
**Name**	**Rodrigues , Larissa**
**Affiliation**	**University of Campinas, Obstetrics & Gynecology**
**Date**	**20-Jan-2025**
**COI**	**None**

Thank you very much for the opportunity to review this manuscript.

Below are some considerations:

Title: I suggest putting the method as a subtitle

Abstract

I suggest reviewing after changes to the manuscript.

Introduction

The paragraphs are very long, making them difficult to read. I suggest breaking them up into smaller paragraphs.

I believe that the aims need to be presented in a more direct and organized way at the end of the introduction section. Any contextualization should come first.

Methods

I believe it is necessary to define the type of study in a section of the method. The authors start by presenting an intervention or data collection strategy (workshop), which becomes confusing.

It is not clear how birth goals compared between workshops and interviews

I understand the importance of supplementary materials, but I believe that the method needs to be better described in the main text.

I noticed that the authors use the SRQR checklist, but since the study included an intervention or quantitative analysis, I think they would need to use a checklist in this regard as well. I make this observation both to better organize the text and to better understand the results and discussion.

What advantages does the sample composed of 3 parts with 3 groups of people in different positions in the childbirth care experience bring to the research? Was it a triangulation? I believe this needs to be better explained.

The issue of data generalization is addressed superficially, I believe that more information could help understanding: how will this generalization be done, with this sample? How will it be possible to make another type of generalization? With what quantity and composition of sample?

The inference I make is that the authors are raising and organizing the problem and therefore it would be the initial phase to develop an intervention to improve issues regarding childbirth for women with high body weight, but this was not clear.

The figures and tables are beautiful and explanatory, but perhaps they could provide more information about the intervention itself.

Research questions

Missing research question

Results, Discussion, Conclusion

I believe it is important to present the results that the authors bring, but I miss the structure mentioned above.

The goals of childbirth care seem to me to be common to all women, considering the literature and clinical practice, in this sense, another important issue would be the discussion of what is specific to women with higher body weight.

Ethical aspects

I did not understand how the study does not fall within the scope of the human research law, I would like to understand what situation it falls within in order to understand the authorization and control of ethical aspects.

VERSION 1 - AUTHOR RESPONSE

Reviewer 1: Miss Larissa Rodrigues, University of Campinas

Comments to Author

Thank you very much for the opportunity to review this manuscript. Below are some considerations:

We are grateful to Reviewer 1, Miss Larissa Rodrigues, for providing feedback on our manuscript.

Title

- I suggest putting the method as a subtitle

We agree with the Reviewer that the title required revision. In response to the Editors' and Reviewer's comments and in accordance with the journal guidelines, we have revised the title:
"Identifying fundamental goals of childbirth care for women with higher body weight in Swiss maternity care: An exploratory multi-stakeholder approach" (p. 1)

Abstract

* I suggest reviewing after changes to the manuscript.

We thank the Reviewer for this valuable suggestion. Following the revisions to the manuscript, we have updated the abstract to reflect the changes (p. 2).

Introduction

* The paragraphs are very long, making them difficult to read. I suggest breaking them up into smaller paragraphs.

We acknowledge the Reviewer's feedback regarding the length and readability of the Introduction. To address this concern, we have revised the structure and language of the Introduction section in order to enhance its clarity and flow while maintaining a scientific tone. Please refer to the main document for the specific revisions (pp. 4f).

* I believe that the aims need to be presented in a more direct and organized way at the end of the introduction section. Any contextualization should come first.

We thank the Reviewer for this valuable input. Since another comment concerning the absence of research questions also pertains to this section (see below), we have addressed both comments together. Accordingly, we have restructured the final paragraphs of the Introduction, beginning with the contextualization, followed by the research questions and the objectives of the study.
"The significance of identifying care goals in healthcare decision-making is well acknowledged[2,3] *as incomplete representations of values and priorities can profoundly affect care decisions and outcomes.*

Involving key stakeholders is thereby consistent with contemporary ethical and moral standards in healthcare[4,5] *and enhances the legitimacy and acceptability of decisions.*[6] *Ongoing multi-stakeholder research aims to establish a core outcome set for trials evaluating interventions in pregnant women with higher body weight.*[7] *However, we found no studies that explicitly examined fundamental child- birth care goals to inform decision-making specifically for women with higher body weight, nor for the general birthing population. Explicit knowledge of such goals could structure the biopsychosocial com- plexities of navigating childbirth care for women with higher body weight and facilitate goal-oriented deliberation throughout decision-making processes.*[6]
For complex decisions with diverse goals, stakeholders may struggle to articulate their goals without support, making it critical to help them identify what is fundamentally important to achieve.[8-10] *Re- search*

by Bond et al.[8,9] *and Haag et al.*[10] *has demonstrated that combining brainstorming with selecting relevant goals from a pre-established masterlist can generate more comprehensive sets of goals in general decision analysis studies and public environmental planning. Notably, even limited numbers of participants have thereby been shown to capture the complete set of goals.*[10] *We hypothesized that these findings may generalize to the healthcare context. While this has not yet been empirically validated, it could inform future efforts in identifying healthcare goals.*

To address these knowledge gaps, the study focused on the following three research questions: (1) What are the fundamental goals of childbirth care for women with higher body weight? (2a) Can a combina- tion of brainstorming and goal selection from a masterlist help stakeholders generate a more diverse set of fundamental goals in a healthcare context? (2b) How many participants are required to fully capture the fundamental goals using this method? Therewith, the first objective of the study was to identify a diverse set of fundamental goals of childbirth care for women with higher body weight to inform decision-making processes by drawing on multi-stakeholder insights. The second objective in- cluded evaluating the masterlist method to support these healthcare stakeholders in generating a more diverse set of fundamental goals." (pp. 4f)

Methods

- I believe it is necessary to define the type of study in a section of the method. The authors start by presenting an intervention or data collection strategy (workshop), which becomes confusing.

We appreciate the Reviewer's comment highlighting the missing information on the research approach and study design. In response, we have restructured the Methods section to include a new paragraph:

"Research approach and study design

Given the limited prior research on our study topic, we employed an exploratory approach using an embedded mixed methods design. The exploratory approach allowed to systematically integrate quali- tative and quantitative methods to gain new insights into fundamental goals of childbirth care for women with higher body weight and to evaluate the masterlist method for supporting goal generation in a healthcare context.[11] *The embedded mixed methods design well-suited our study, as the interrelated research questions required the combination of different but complementary types of data.*[12] *To identify goals considered fundamentally important for achieving the best possible childbirth care, we conducted group workshops and individual interviews with key stakeholders. This qualitative process was guided by value-focused thinking according to Keeney.*[13] *We aimed to uncover the stakeholders' underlying values and priorities, rather than centering decision-making on selecting among predefined options, as in conventional "alternative-focused thinking".*[13] *The quantitative component, which assessed the ef- fectiveness of the masterlist method, was embedded within the qualitative process.*[12] *While each com- ponent addressed separate research questions, the quantitative evaluation was inherently dependent on the qualitative identification of the fundamental care goals.*[12] *The embedded mixed methods design therefore facilitated a better understanding of both the fundamental goals of childbirth care for women with higher body weight*

and the methodological utility for generating diverse healthcare goals." (pp. 5f)

* It is not clear how birth goals compared between workshops and interviews

The first objective of this study was to identify a diverse set of fundamental goals of childbirth care for women with higher body weight to inform decision-making processes by drawing on multi-stakeholder insights. However, the study was not designed to compare the goals identified in group workshops with those from individual interviews, and no formal comparative analysis was conducted. As such, this aspect has not been addressed in the manuscript.

In line with our second objective of evaluating the masterlist method to support stakeholders in generating more diverse goals, we examined how many goals individual participants self-generated during brain-storming and how many additional new goals they selected (i.e., recognized) from the masterlist. This analysis was possible because the first three steps of data collection were identical in both group workshops and individual interviews, before participants engaged in group discussions or individual elaboration of their goals (Table 1, p. 9). We hope that this clarification satisfactorily addresses the Reviewer's concern.

* I understand the importance of supplementary materials, but I believe that the method needs to be better described in the main text.

We agree that a more detailed description of the methods in the main text enhances the transparency of our research process, particularly regarding the masterlist method used to support participants in generating a more diverse set of goals and the evaluation of this method. To address this concern, we have incorporated key methodological details from the Supplementary Materials into the main text. Due the extent of these revisions, we kindly refer to the main document for an overview of the changes (e.g., pp. 7, 10f).

* I noticed that the authors use the SRQR checklist, but since the study included an intervention or quan-titative analysis, I think they would need to use a checklist in this regard as well. I make this observation both to better organize the text and to better understand the results and discussion.

We appreciate the Reviewer's observation regarding the use of an appropriate reporting checklist for the study. The SRQR checklist was applied specifically to the first part of the study, which focused on identi-fying fundamental goals of childbirth care for women with higher body weight. This part of the study aligns primarily with qualitative healthcare research, albeit in a less conventional sense.

In the second part of the study, which involved evaluating the masterlist method to support stakeholders in generating more diverse goals, the focus shifted from healthcare research to the application of decision analysis within a healthcare context. We kindly ask the Reviewer to take this shift into account when assessing our work. Due to this change in focus, we were unable to identify a reporting checklist within the EQUATOR network that appropriately aligns with this type of analysis. Although the CReDECI 2 checklist[14] was considered, we concluded that our decision analysis approach does not conform to the characteristics of complex healthcare interventions typically addressed by this framework. Therefore, we

decided against forcing our approach into this structure.
However, in response to this Reviewer's comment and others, we have made efforts to improve the structure, clarity, and transparency of the manuscript to enhance its comprehensibility for readers.

* What advantages does the sample composed of 3 parts with 3 groups of people in different positions in the childbirth care experience bring to the research? Was it a triangulation? I believe this needs to be better explained.

We thank the Reviewer for allowing us to clarify this important methodological part. Individuals' roles in decision-making may strongly influence their goal priorities.[cf.15] Accordingly, we identified women with higher body weight (preconceptional BMI $\geq 30 kg/m^2$), midwives, and obstetricians as key stakeholders directly involved in childbirth care decisions. Their fundamental goals should explicitly be considered when evaluating appropriate care from diverse perspectives, while the final decisions should be made by the women themselves.
Although the distinction between these three stakeholder groups in our study results was not part of a formal triangulation process, it was initially established to explore potential variations in goal generation and valuation. However, due to the small sample sizes for each group (ranging from five to nine partici- pants), we decided not to report between-group comparisons.
To avoid unintended comparative interpretations and improve clarity, we have revised the manuscript, tables, and Supplementary Materials, removing this distinction. The only exception is the table on participants' characteristics, which provides an overview of the stakeholders involved (p. 11). We hope these revisions enhance the clarity of our research.

* The issue of data generalization is addressed superficially, I believe that more information could help understanding: how will this generalization be done, with this sample? How will it be possible to make another type of generalization? With what quantity and composition of sample?

We appreciate the Reviewer's thoughtful comment on the issue of generalization. This study does not claim that the identified goals are universal or equally important to all women with higher body weight and their maternity care providers. Rather, it aims to present a set of diverse potential goals of childbirth care from which individuals can select and prioritize those most relevant to their unique decision-making situations.
Our saturation analysis indicated that a theoretical maximum of six participants would have been sufficient to capture the complete set of fundamental goals in our study, with no further fundamental goals emerging from the additional 15 participants. Thus, increasing the sample size would probably not have yielded greater diversity in goals. However, recognizing variations in individual preferences, circumstances, and care settings, we acknowledge that some identified goals may not be valued by every individual, and additional goals may emerge in different contexts. While the identified set of goals is not intended to be comprehensive or universally applicable, our primary findings may provide a useful framework for informed deliberation in contexts similar to our study.

Future studies could validate and expand this framework of fundamental goals to include diverse socioeconomic and -cultural contexts, different healthcare systems, and populations, such as the general birthing population or women with other complexities. This would allow for encompassing goals that may not have been captured within the specific context of Swiss maternity care for women with higher body weight.

In response to this comment, we have revised the section on strengths, limitations, and research needs to clarify these points and more thoroughly address the transferability of the findings (pp. 18f).

* The inference I make is that the authors are raising and organizing the problem and therefore it would be the initial phase to develop an intervention to improve issues regarding childbirth for women with high body weight, but this was not clear.

Indeed, the intention of this study was to establish a framework for structuring and informing decision-making on tailored, high-quality childbirth care for women with higher body weight. We appreciate the Reviewer's observation regarding the clarity of this point. To address this concern, we have emphasized the relevant information in the Abstract, Introduction, Discussion, and Conclusions sections of the manuscript. For example:

Abstract:

"The first objective of the study was to identify fundamental goals of childbirth care for women with higher body weight to inform decision-making processes, drawing on multi-stakeholder insights." (p. 2)

"The fundamental goals provide a framework for benchmarking decisions to improve childbirth care for women with higher body weight." (p. 2)

Introduction section:

"Identifying and understanding such fundamental goals provides a comprehensive framework for selecting the best possible childbirth care option in a given decision situation." (p. 4)

"Explicit knowledge of such goals could structure the biopsychosocial complexities of navigating childbirth care for women with higher body weight and facilitate goal-oriented deliberation throughout decision-making processes.[6]" (p. 5)

Discussion section:

"This fundamental goal set may encourage value-focused deliberation throughout decision-making processes and serve as a framework for finding the most appropriate option to ensure tailored, high- quality childbirth care." (p. 14)

"Explicitly addressing multidimensional goals in decision-making may therefore be particularly essen- tial for improving childbirth care and outcomes for women with higher body weight from a biopsycho- social health and resource sustainability perspective." (p. 14)

"Aligning decisions with the fundamental goals generated in a value-focused process offers an opportunity to promote childbirth care that is safe, person-centered, sustainable, and responsive to the needs and preferences of women with higher body weight." (p. 16)

"To advance childbirth care for women with higher body weight, ongoing research is needed to eval- uate

the utility of the fundamental goals using methods such as Multi-Criteria Decision Analysis (MCDA).[16,17]" (p. 19)

Conclusions section:

"By providing empirical evidence on multidimensional goals, [the study] establishes a framework for facilitating decision-making on childbirth care. Moreover, the set of fundamental goals may serve as a benchmark for evaluating and improving childbirth services. Our approach thereby contributes to a better understanding and promotes discourse about what is fundamentally valued from the perspectives of multiple key stakeholders." (p. 19)

- The figures and tables are beautiful and explanatory, but perhaps they could provide more information about the intervention itself.

We thank the Reviewer for this positive feedback. In line with our response to the third comment on the Methods section, we have integrated key details about the masterlist method from the Supplementary Materials into the main text and revised the tables where possible to enhance clarity and comprehensiveness. Given the extent of these revisions, we kindly refer to the main document for an overview of the changes (e.g., p. 7).

Research questions

- Missing research question

We appreciate the Reviewer for highlighting the missing research questions. As outlined in our response to a previous comment, we have added our three research questions to the final paragraph of the Introduction section, followed by the study objectives:

"To address these knowledge gaps, the study focused on the following three research questions: (1) What are the fundamental goals of childbirth care for women with higher body weight? (2a) Can a combination of brainstorming and goal selection from a masterlist help stakeholders generate a more diverse set of fundamental goals in a healthcare context? (2b) How many participants are required to fully capture the fundamental goals using this method? Therewith, the first objective of the study was to identify a diverse set of fundamental goals of childbirth care for women with higher body weight to inform decision-making processes by drawing on multi-stakeholder insights. The second objective in- cluded evaluating the masterlist method to support these healthcare stakeholders in generating a more diverse set of fundamental goals." (p. 5)

Results, Discussion, Conclusion

- I believe it is important to present the results that the authors bring, but I miss the structure mentioned above.

We thank the Reviewer for emphasizing the importance of a clear and coherent presentation of our findings. In response to this concern, we have refined the structure of the Results section by resolving incon-

sistencies and incorporating subtitles according to the research questions to improve clarity and coherence. We hope these adjustments further enhance the organization of the manuscript. For a detailed overview of the changes, please refer to the main document (pp. 11 to 13).

* The goals of childbirth care seem to me to be common to all women, considering the literature and clinical practice, in this sense, another important issue would be the discussion of what is specific to women with higher body weight.

The Reviewer raises an important point that warrants careful consideration. We acknowledge that many of the childbirth care goals identified in this study are likely relevant to all women and their maternity care providers, regardless of body weight or case complexity. This aligns with both existing literature and own clinical experience. However, we found no studies explicitly examining fundamental childbirth care goals to inform decision-making – neither for the general birthing population nor specifically for women with higher body weight. As our study focused on the latter, we cannot draw broader conclusions beyond this population.

To address this important point, we have complemented the Introduction section and revised the Discussion section to further emphasize why the identified goals may be particularly relevant for women with higher body weight. This includes considerations related to their increased likelihoods of obstetric complications, medical interventions, adverse biopsychosocial outcomes, and higher resource utilization.[18-25] Given this evidence, integrating multidimensional goals into decision-making could be essential for improving childbirth care and outcomes in this population. We appreciate the Reviewer's valuable feedback and kindly refer to the main document for the specific revisions (e.g., pp. 5, 14, 16).

Ethical aspects

* I did not understand how the study does not fall within the scope of the human research law, I would like to understand what situation it falls within in order to understand the authorization and control of ethical aspects.

We appreciate that the Reviewer draws attention to the lack of clarity on ethical aspects. In Switzerland, the Human Research Act regulates research involving human subjects, aiming to protect their dignity, privacy, and health.[26] The scope of this Act encompasses studies on human diseases, as well as on the structure and function of the human body. It applies to research involving living or deceased individuals, embryos and fetuses, biological materials, and health-related personal data.[26]

To assess the applicability of the Swiss Human Research Act[26] to our study, we submitted a clarification of responsibility to the Ethics Committee for Research of the Canton of Berne, Switzerland. After reviewing the research project, the committee concluded that the collection and analysis of individual and professional care goals from stakeholders, along with their underlying values and priorities, does not constitute health-related personal data as defined by the Act. Consequently, the committee waived the requirement for formal ethical approval (decision BASEC-No. Req-2019-00531).

To improve clarity in the manuscript, we have revised the corresponding paragraph in the Methods section accordingly:

"The Ethics Committee for Research of the Canton of Berne, Switzerland, declared that our study does not fall under the Swiss Human Research Act (2011), Art. 2, para. 1.[26] *The committee concluded that the collection and analysis of care goals based on the underlying values and priorities of stakeholders does not constitute health-related personal data as defined by the Act. Consequently, formal ethical approval was waived (decision BASEC-No. Req-2019-00531)." (p. 6)*

References

1. Beutler P, Larsen TA, Maurer M, Staufer P, Lienert J. A participatory multi-criteria decision analysis framework reveals transition potential towards non-grid wastewater management. *J Environ Manage*. 2024;367. doi:10.1016/j.jenvman.2024.121962
2. Australian Commission on Safety and Quality in Health Care [ACSQHC]. *Implementing the comprehensive care standard: identifying goals of care*. 2019. https://www.safetyandquality.gov.au/sites/default/files/2019-06/implementing-the-comprehensive-care-standard-identifying-goals-of-care-final-accessible-pdf-24-apr-2019.pdf
3. Elwyn G, Vermunt NPCA. Goal-based shared decision-making: developing an integrated model. *J Patient Exp*. 2020;7(5):688-696. doi:10.1177/2374373519878604
4. Institute of Medicine, Committee on Quality of Health Care in America. *Crossing the quality chasm: a new health system for the 21st century*. National Academies Press, US; 2001.
5. International Federation of Gynecology and Obstetrics [FIGO]. *Ethical framework for respectful maternity care during pregnancy and childbirth*. 2021. https://www.figo.org/sites/default/files/2021-09/FIGO_Statement_Ethical-Framework-Respectful-Maternity-Care-During-Pregnancy-Childbirth_0.pdf
6. Goetghebeur M, Wagner M. Identifying value(s): a reflection on the ethical aspects of MCDA in healthcare decisionmaking. In: Marsh K, Goetghebeur M, Thokala P, Baltussen R, eds. *Multi-criteria decision analysis to support healthcare decisions*. Springer; 2017:29-46.
7. Dadouch R, Faheim M, Juando-Prats C, Parsons J, D'Souza R. Development of a core outcome set for studies on obesity in pregnant patients (COSSOPP): a study protocol. *Trials*. 2018;19(1):655. doi:10.1186/s13063-018-3029-1
8. Bond SD, Carlson KA, Keeney RL. Generating objectives: can decision makers articulate what they want? *Manag Sci*. 2008;54(1):56-70.
9. Bond SD, Carlson KA, Keeney RL. Improving the generation of decision objectives. *Decis Anal*. 2010;7(3):238-255. doi:10.1287/deca.1100.0172
10. Haag F, Zürcher S, Lienert J. Enhancing the elicitation of diverse decision objectives for public planning. *Eur J Oper Res*. 2019;279(3):912-928. doi:10.1016/j.ejor.2019.06.002
11. Stebbins RA. *Exploratory research in the social sciences*. SAGE; 2001.
12. Creswell JW, Plano Clark VL. *Designing and conducting mixed methods research*. 2nd ed. SAGE; 2011.
13. Keeney RL. *Value-focused thinking: a path to creative decisionmaking*. Harvard University Press;

1992.

14. Möhler R, Köpke S, Meyer G. Criteria for Reporting the Development and Evaluation of Complex Interventions in healthcare: revised guideline (CReDECI 2). *Trials*. May 3 2015;16:204. doi:10.1186/s13063-015-0709-y

15. Marttunen M, Belton V, Lienert J. Are objectives hierarchy related biases observed in practice? A meta-analysis of environmental and energy applications of Multi-Criteria Decision Analysis. *Eur J Oper Res*. 2018;265(1):178-194. doi:10.1016/j.ejor.2017.02.038
16. Eisenführ F, Weber M, Langer T. *Rational decision making*. Springer; 2010.
17. Thokala P, Devlin N, Marsh K, et al. Multiple criteria decision analysis for health care decision making - An introduction: report 1 of the ISPOR MCDA Emerging Good Practices Task Force. *Value Health*. 2016;19(1):1-13. doi:10.1016/j.jval.2015.12.003
18. D'Souza R, Horyn I, Pavalagantharajah S, Zaffar N, Jacob CE. Maternal body mass index and pregnancy outcomes: a systematic review and metaanalysis. *Am J Obstet Gynecol MFM*. 2019;1(4):100041. doi:10.1016/j.ajogmf.2019.100041
19. Creanga AA, Catalano PM, Bateman BT. Obesity in pregnancy. *N Engl J Med*. 2022;387(3):248-259. doi:10.1056/NEJMra1801040
20. Vats H, Saxena R, Sachdeva MP, Walia GK, Gupta V. Impact of maternal pre-pregnancy body mass index on maternal, fetal and neonatal adverse outcomes in the worldwide populations: a systematic review and meta-analysis. *Obes Res Clin Pract*. 2021;15(6):536-545. doi:10.1016/j.orcp.2021.10.005
21. Kalliala I, Markozannes G, Gunter MJ, et al. Obesity and gynaecological and obstetric conditions: umbrella review of the literature. *BMJ*. 2017;359:j4511. doi:10.1136/bmj.j4511
22. Thorbjörnsdottir KE, Karlsen IE, Dahl B, Røseth I. “Talk to me, not at me”: obese women’s experiences of birth and their encounter with birth attendants - a qualitative study. *Int J Qual Stud Health Well-being*. 2020;15(1):1845286. doi:10.1080/17482631.2020.1845286
23. Incollingo Rodriguez AC, Smieszek SM, Nippert KE, Tomiyama AJ. Pregnant and postpartum women’s experiences of weight stigma in healthcare. *BMC Pregnancy Childbirth*. 2020;20(1):499. doi:10.1186/s12884-020-03202-5
24. Moran PS, Wuytack F, Turner M, et al. Economic burden of maternal morbidity: a systematic review of cost-of-illness studies. *PloS one*. 2020;15(1):e0227377. doi:10.1371/journal.pone.0227377
25. Marchi J, Berg M, Dencker A, Olander EK, Begley C. Risks associated with obesity in pregnancy, for the mother and baby: a systematic review of reviews. *Obes Rev*. 2015;16(8):621-38. doi:10.1111/obr.12288
26. Swiss Confederation (Schweizerische Eidgenossenschaft). Federal act on research involving human beings (Human Research Act, HRA). 2023. https://www.fedlex.admin.ch/eli/cc/2013/617/en
